# Sea-Island-Like Morphology of CuNi Bimetallic Nanoparticles Uniformly Anchored on Single Layer Graphene Oxide as a Highly Efficient and Noble-Metal-Free Catalyst for Cyanation of Aryl Halides

**DOI:** 10.1038/s41598-020-57483-z

**Published:** 2020-01-20

**Authors:** Gopiraman Mayakrishnan, Vijayakumar Elayappan, Ick Soo Kim, Ill-Min Chung

**Affiliations:** 1 Department of Crop Science, College of Sanghur Life Science, Konkuk University, 120 Neungdong-ro, Gwangjin-gu, Seoul 05029 South Korea; 2 Department of Materials Science and Technology, Korea University, Seoul, 02841 South Korea; 3 Nano Fusion Technology Research Group, Division of Frontier Fibers, Institute for Fiber Engineering (IFES), Interdisciplinary Cluster for Cutting Edge Research (ICCER), Shinshu University, Tokida 3-15-1, Ueda, Nagano Prefecture 386-8567 Japan

**Keywords:** Materials science, Materials for energy and catalysis, Nanoscale materials

## Abstract

Aryl nitriles are versatile compounds that can be synthesized *via* transition-metal-mediated cyanation of aryl halides. Most of the supported-heterogeneous catalysts are noble-metals based and there are very limited numbers of efficient non-noble metal based catalysts demonstrated for the cyanation of aryl halides. Herein, bimetallic CuNi-oxide nanoparticles supported graphene oxide nanocatalyst (CuNi/GO-I and CuNi/GO-II) has been demonstrated as highly efficient system for the cyanation of aryl halides with K_4_[Fe(CN)_6_] as a cyanating agent. Metal-support interaction, defect ratio and synergistic effect with the bimetallic nanocatalyst were investigated. To our delight, the CuNi/GO-I system activity transformed a wide range of substrates such as aryl iodides, aryl bromides, aryl chlorides and heteroaryl compounds (Yields: 95–71%, TON/TOF: 50–38/2 h^−1^). Moreover, enhanced catalytic performance of CuNi/GO-I and CuNi/GO-II in reduction of 4-nitropehnol with NaBH_4_ was also confirmed (k_app_ = 18.2 × 10^−3^ s^−1^ with 0.1 mg of CuNi/GO-I). Possible mechanism has been proposed for the CuNi/GO-I catalyzed cyanation and reduction reactions. Reusability, heterogeneity and stability of the CuNi/GO-I are also found to be good.

## Introduction

**Figure 1:**
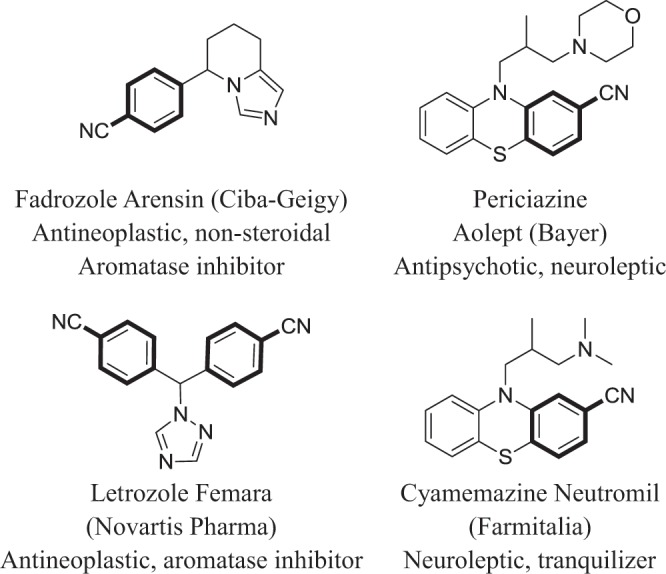
Examples of pharmaceutically important benzonitriles.

Nitriles are versatile building blocks in the synthesis of numerous commercial compounds including natural products, agrochemicals (herbicides and pesticides), and pharmaceuticals^1,2^. For instance, some of the pharmaceutically important benzonitriles are provided in Fig. 1. Traditional synthesis of aromatic nitriles is highly limited due to the use of stoichiometric toxic cyanide agents at elevated temperatures, and often requires complicated workups^3,4^. Catalytic cyanation of aromatic aryl halides is one of the graceful methods to access such aromatic nitriles. Most of the developed methods are transition metals (particularly Pd, Cu and Ni) based homogenous catalytic systems^1,5–7^. Zhang *et al*.^8^, used Cu(OAc)_2_ as a catalyst for the cyanation of aryl halide with cyanide source (combination of NH_4_HCO_3_ and HCON(CH_3_)_2_). Alike, NiCl_2_·6H_2_O/dppf/Zn catalytic system was developed for the cyantion of hetero(aryl) chlorides by Zhang and coworkers^9^. Due to practical advantages such as reusability and simple cost-effective workup, heterogeneous catalysts are also developed. Unfortunately, very few numbers of heterogonous catalysts are reported to date which may be due to their poor catalytic performance. In addition, unlike homogenous catalysis, all the reported heterogonous catalysts are noble-metal based systems. For example, Mondal *et al*.^10^, prepared molecular cage impregnated Pd-nanoparticles for cyanation of aryl halides with K_4_[Fe(CN)_6_]. Alike, Kumar *et al*.^11^, found that Pd nanoparticles embedded C@Fe_3_O_4_ core−shell hybrid nanospheres was highly efficient and reusable toward the cyanation of aryl halides. Nanocatalyst, Pd/CuO, which composed of both noble and non-noble metals, was reported for the cyanation of aryl halides under ligand-free conditions^12^. To overcome the above mentioned drawbacks, Nasrollahzadeh *et al*.^13^, prepared a simple CuO-nanoparticles supported carbon catalysts (CuO/C) for the cyanation of aryl halides; however, the CuO/C is inert towards the aryl bromides and chlorides. Although number of cost-effective Cu and Ni mediated catalytic system are developed, to the best of our knowledge, there is no efficient and stable non-noble metals based heterogeneous catalyst reported for the cyanation reaction to date. Hence, development of highly efficient and cost-effective non-noble metal based catalysts, particularly based on Cu and Ni, is highly demanding one.

Nanostructured transition metals (such as Cu, Ni, Ag, Au, Pd and Pt) supported graphene nanocomposites have found to be highly efficient candidates for a number of industrially important catalytic processes^14,15^. Astonishing physicochemical properties such as elegant morphology, stability and huge specific surface make graphene nanocomposites highly suitable for the heterogeneous catalysis^16^. Particularly, mechanochemically prepared carbon nanocomposites showed enhanced catalytic performance which is mainly due to the better metal-support interaction^17–20^. For instance, inert RuO_2_-nanoparticles were tuned to highly efficient catalyst by decorating it onto single-walled carbon nanotubes (SWCNTs) by a simple ‘dry synthesis’^19^. The resultant RuO_2_/SWCNTs demonstrated excellent catalytic performance in Heck olefination of aryl halides. Similarly, CuO-graphene nanocatalyst prepared by mechanochemical synthesis was found to be highly efficient for the A^3^-coupling and *aza*-Michael reactions^21^. In fact, oxygen functional groups and carbon vacancies in graphene have capability to interact with metal clusters which could tune the inactive metals to be an active catalyst. Hence, we presumed that bimetallic Cu-Ni supported graphene oxide catalyst would be an alternate choice for the noble-metals supported heterogeneous catalysts. Moreover, bimetallic nanoparticles provide stability and functionality comparable to noble metals at a lower cost. Herein, we prepared bimetallic CuNi-oxide nanoparticles supported graphene nanocatalysts (CuNi/GO-I and CuNi/GO-II) by a very simple mechanochemical synthesis. The prepared nanocatalysts were characterized well by using high resolution transmission electron microscope (HR-TEM), atomic force microscope (AFM), inductively coupled plasma-mass spectroscope (ICP-MS), energy dispersive spectroscope (EDS), scanning electron microscope (SEM), X-ray diffraction (XRD), X-ray photoemission spectroscope (XPS), Brunauer-Emmett-Teller (BET) and Raman. After being optimized, the nanocatalysts were used for the cyanation of aryl halides. In addition, the nanocatalysts were also used for the reduction of 4-nitrophenol with NaBH_4_ in order to investigate its versatility. In fact, the catalytic reduction of 4-nitrophenol is very significant reaction in green chemistry and the product (4-aminophenols) is often used in the preparation of synthetic dyes, herbicides and pesticides^22,23^. Heterogeneity, reusability, and stability of the nanocatalysts were tested. Possible mechanism for the cyanation and reduction reactions is discussed.

## Experimental Section

### Materials

Reduced graphene oxide (GO, thickness ~ ≤ 3.0 nm, surface area ~ > 600 m^2^/g and purity ~ > 99 wt %) was supplied by ACS Materials (USA) and used without any further purification. Metal precursors, Ni(acac)_2_ (97%) and Cu(acac)_2_, were purchased from Sigma Aldrich (USA). Potassium ferrocyanide (K_4_Fe(CN)_6_), sodium borohydride (NaBH_4_) aryl halides, solvents and 4-nitrophenol were supplied by Sigma Aldrich (USA) or Wako Pure Chemicals (Japan), and used as received.

### Preparation of bimetallic CuNi/GO nanocatalysts

**Scheme 1:**
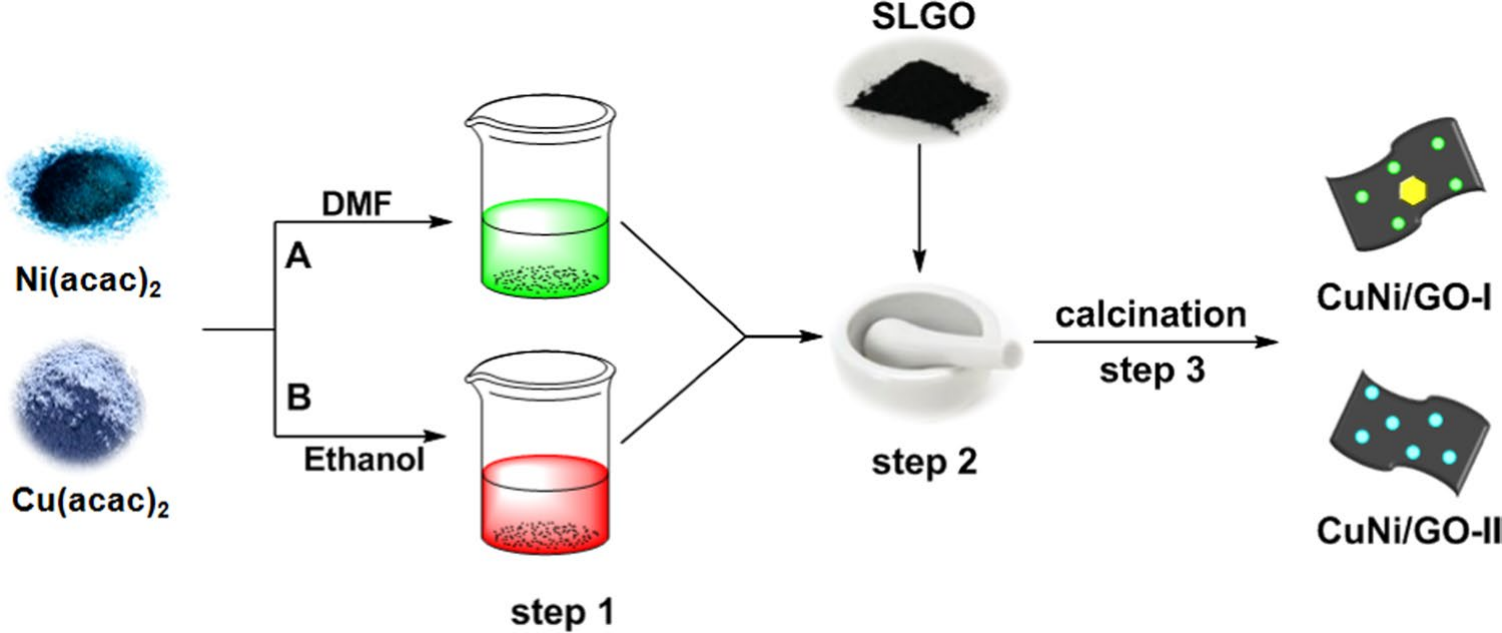
Schematic illustration for the preparation of CuNi/GO-I and CuNi/GO-II.

Reducing agent and capping agent free mechanochemical preparation method was adopted for the preparation of nanocatalysts, CuNi/GO-I and CuNi/GO-II. In a typical preparation of CuNi/GO-I, a mixture of GO (500 g), DMF (25 mL), Ni(acac)_2_ (200 mg) and Cu(acac)_2_ (72 mg) was prepared and heated at 100 °C under vigorous stirring condition for 2 h. Subsequently, DMF was slowly evaporated from the above mixture and a clay-form of GO/Cu(acac)_2_/Ni(acac)_2_ mixture was obtained. The resultant mixture was pestle-grinded for 1 h to achieve a homogenous distribution of Cu(acac)_2_/Ni(acac)_2_ with GO and then the mixture was completely dried under *vacuum*. Finally, the mixture was calcinated under argon atmosphere at 320 °C for 2 h. Similarly, CuNi/GO-II was prepared by using GO (500 g), ethanol (25 mL), Ni(acac)_2_ (200 mg) and Cu(acac)_2_ (72 mg). Schematic illustration of the preparation of nanocatalysts is given in Scheme 1. For comparison, mono metallic Ni/GO-I and Cu/GO-I were also prepared and tested (See Figs. S1–S7 in supporting information).

### Characterization

HRTEM (JEOL JEM*-*2100F) and AFM (Park System model XE100 AFM) were employed to study the surface morphology of CuNi/GO-I and CuNi/GO-II. HRTEM was operated at accelerating voltage of 200 kV and a non-contact mode was opted to recode the AFM imaging. Loading of Cu and Ni was found out by recording SEM-EDS (Hitachi 3000 H SEM). To study the metal-support interaction and crystalline properties, Raman spectra (Hololab 5000, Kaiser Optical Systems Inc., USA) and powder XRD (Rotaflex RTP300 (Rigaku Co., Japan) were recorded for CuNi/GO-I and CuNi/GO-II. XPS spectra (Kratos Axis-Ultra DLD, Kratos Analytical Ltd, Japan) were recorded for fresh GO, CuNi/GO-I and CuNi/GO-II. Surface area, pore size and pore volume of the fresh GO, CuNi/GO-I and CuNi/GO-II were calculated by BET method (BELSORP-max; BEL Japan, Inc.). Catalytic performance of CuNi/GO-I and CuNi/GO-II towards reduction of nitropehnol was studied by using Ultraviolet-visible (UV-vis, Shimadzu UV-2600 spectrophotometer). Yield of the catalytic products were determined by Gas chromatograph (GC, Shimadzu-2010 gas chromatograph). Nuclear magnetic resonance (NMR) spectra were recorded on a 400 MHz Bruker spectrometer in CDCl_3_ using tetramethylsilane (TMS) as a standard.

### Procedure for cyanation of aryl halides

A mixture of 1,4-dibromobenzene (236 mg, 1.00 mmol), K_4_[Fe(CN)_6_]·3H_2_O (73 mg, 0.17 mmol), K_2_CO_3_ (166 mg, 1.2 mmol), CuNi/GO-I (15 mg, 0.92 mol% of Cu and 0.95 mol% of Ni) and DMF (10 mL) was magnetically stirred under N_2_ atmosphere at 120 °C for 24 h. After completion of the reaction, the CuNi/GO-I was separated out and washed will with diethyl ether to check its reusability. The filtrate was then extracted ethyl acetate and saturated aqueous sodium hydrogen carbonate solutions. The organic layer was dried over anhydrous Na_2_SO_4_. 1,4-dicyanobenzene - Yield: 93%; ^1^H NMR (CDCl_3_, 500 MHz): d 7.80 (s, 4 H). ^13^C NMR (CDCl_3_, 125 MHz): d 116.7, 116.9, 132.7.

### Procedure for reduction of 4-nitrophenol

A mixture of 80 μL of 0.01 M 4-nitrophenol and 4 mL of 0.015 M NaBH_4_ was magnetically stirred under open air atmosphere at 27 °C. After 30 sec of stirring, a 0.1 mg of CuNi/GO-I or CuNi/GO-II was introduced into the above mixture and continued stirring for several minutes. In order to monitor the reaction, UV-vis spectra were recovered (range 250–700 nm) for the reaction mixture at regular time intervals of every 30 sec.

## Results and Discussion

### Characterization of CuNi/GO nanocomposites

**Figure 2:**
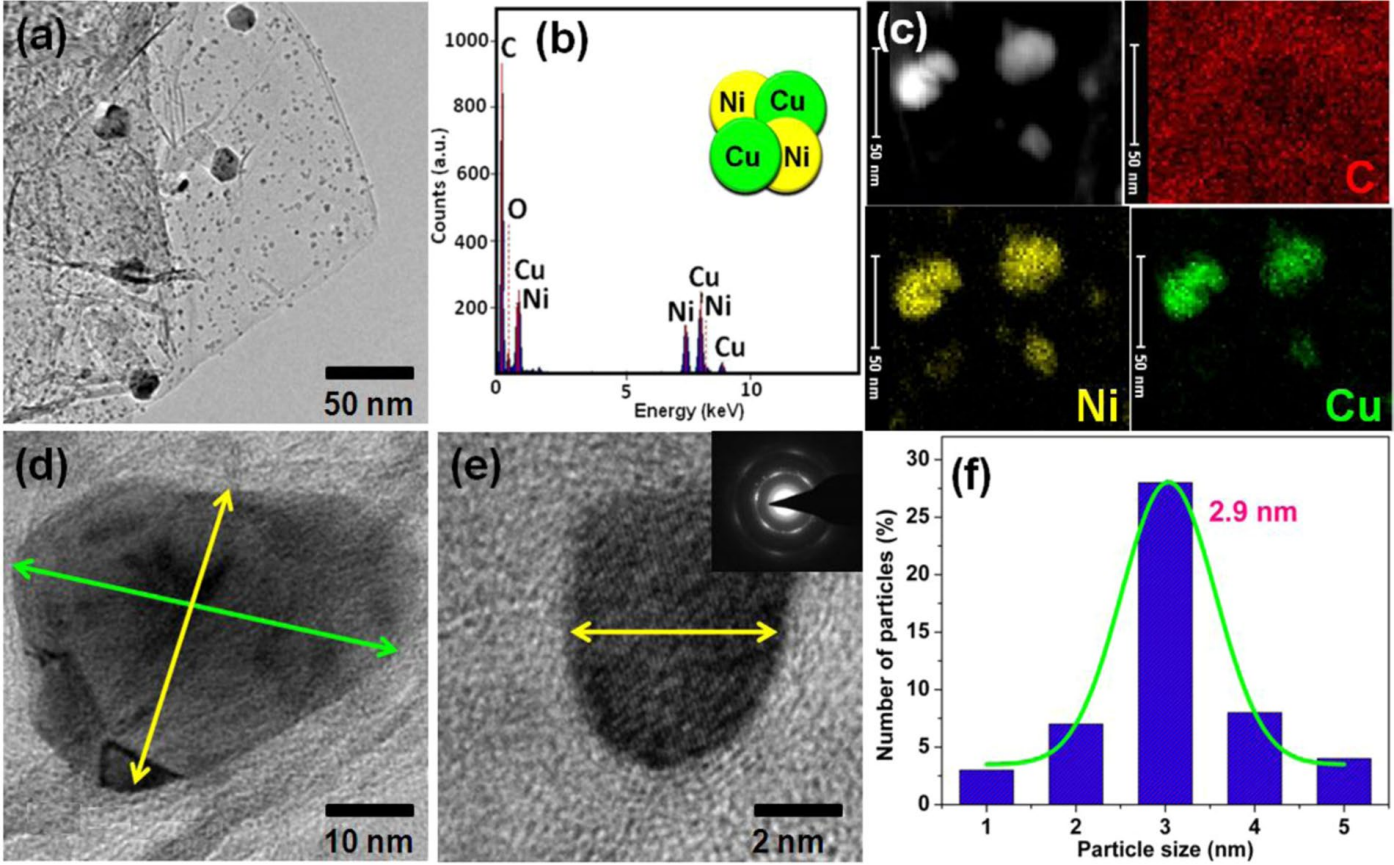
(**a**,**d**,**e**) HR-TEM images of CuNi/GO-I (the inset shows the SAED pattern). (**b**) EDS spectrum of CuNi/GO-I. (**c**) Elemental mapping of C, Ni and Cu for CuNi/GO-I. (**f**) Particle-size distribution histogram of Cu and Ni nanoparticles in CuNi/GO-I.

**Figure 3:**
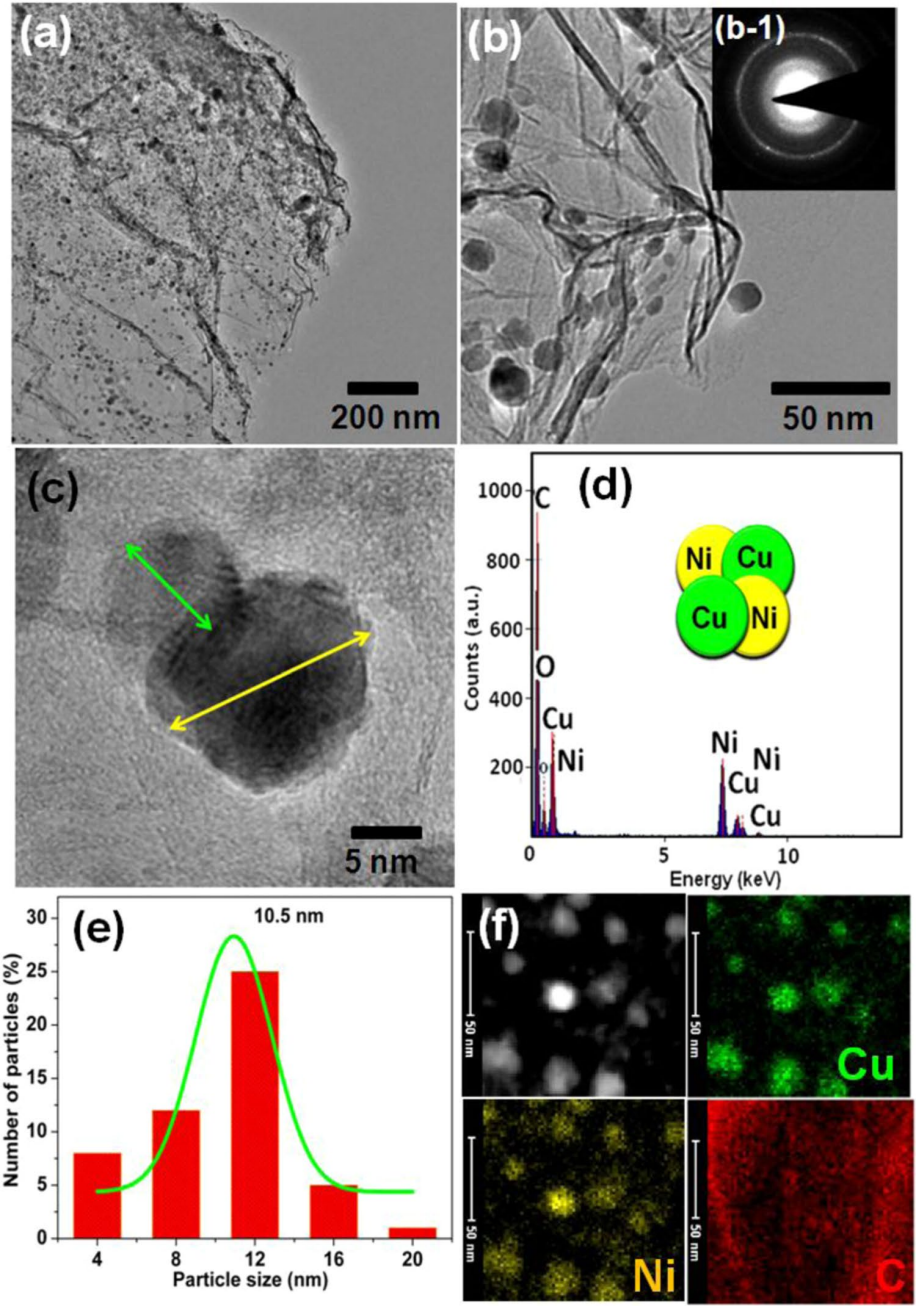
(**a**–**c**) HR-TEM images of CuNi/GO-II (the inset shows the SAED pattern). (**d**) EDS spectrum of CuNi/GO-II. (**e**) Particle-size distribution histogram of Cu and Ni nanoparticles in CuNi/GO-I. (**f**) Elemental mapping of C, Ni and Cu for CuNi/GO-I.

Very simple two step ‘mix and heat’ method using DMF or ethanol was developed for the preparation of bimetallic CuNi/GO-I and CuNi/GO-II catalysts. Figures 2(a–e) and 3(a–d,f) show the HRTEM images, SAED pattern, EDS spectrum and corresponding elemental mapping images of CuNi/GO-I and CuNi/GO-II. Surprisingly, the HRTEM images of CuNi/GO-I confirmed a sea-island like-morphology of CuNi-oxide bimetallic nanoparticles with GO (where sea represents small-size CuNi-oxide nanoparticles with GO, whereas, island represents big CuNi-oxide nanoparticles) (Fig. 2a,d,e). On contrary, the surface morphology of CuNi/GO-II showed no such difference in the particle size, however, the size of CuNi-oxide was found to be quite uniform (Fig. 3a–c). The surprising difference in the morphology of the CuNi/GO catalysts is may be due to the difference in boiling point of solvents since it could play crucial role in the recrystallization of metal salts during the solvent evaporation (Scheme 1). The average size of CuNi-oxide nanoparticles was calculated to be 2.9 nm for CuNi/GO-I and 10.5 nm for CuNi/GO-II (Figs. 2(f) and 3(e)). It can be noted that the average particle size of the CuNi-oxide is 3 times lower for CuNi/GO-I when compared to CuNi/GO-II. The EDS spectrum of CuNi/GO-I showed the presence of C, O, Cu and Ni in 90.7, 1.7, 3.7 and 3.9 wt%, respectively. Similarly, the content of C, O Cu and Ni in CuNi/GO-II was determined to be 91.1, 1.6, 3.5 and 3.8 wt%, respectively. The mapping images showed that the Cu and Ni are well-alloyed and uniformly anchored on the surface of GO. The SAED patters confirmed a polycrystalline nature of the CuNi-oxide nanoparticles (Figs. 2e and 3(b-1)). The lattice constants obtained from the HRTEM analysis were not consistent with either a pure Cu or Ni phase, indicating the formation of well-alloyed CuNi-oxide nanoparticles^24–26^. Other ring patterns may be due to the combination of Cu and Ni oxides (combination of two or more forms - Cu_2_O, NiO, Ni_2_O_3_, or CuO).

**Figure 4:**
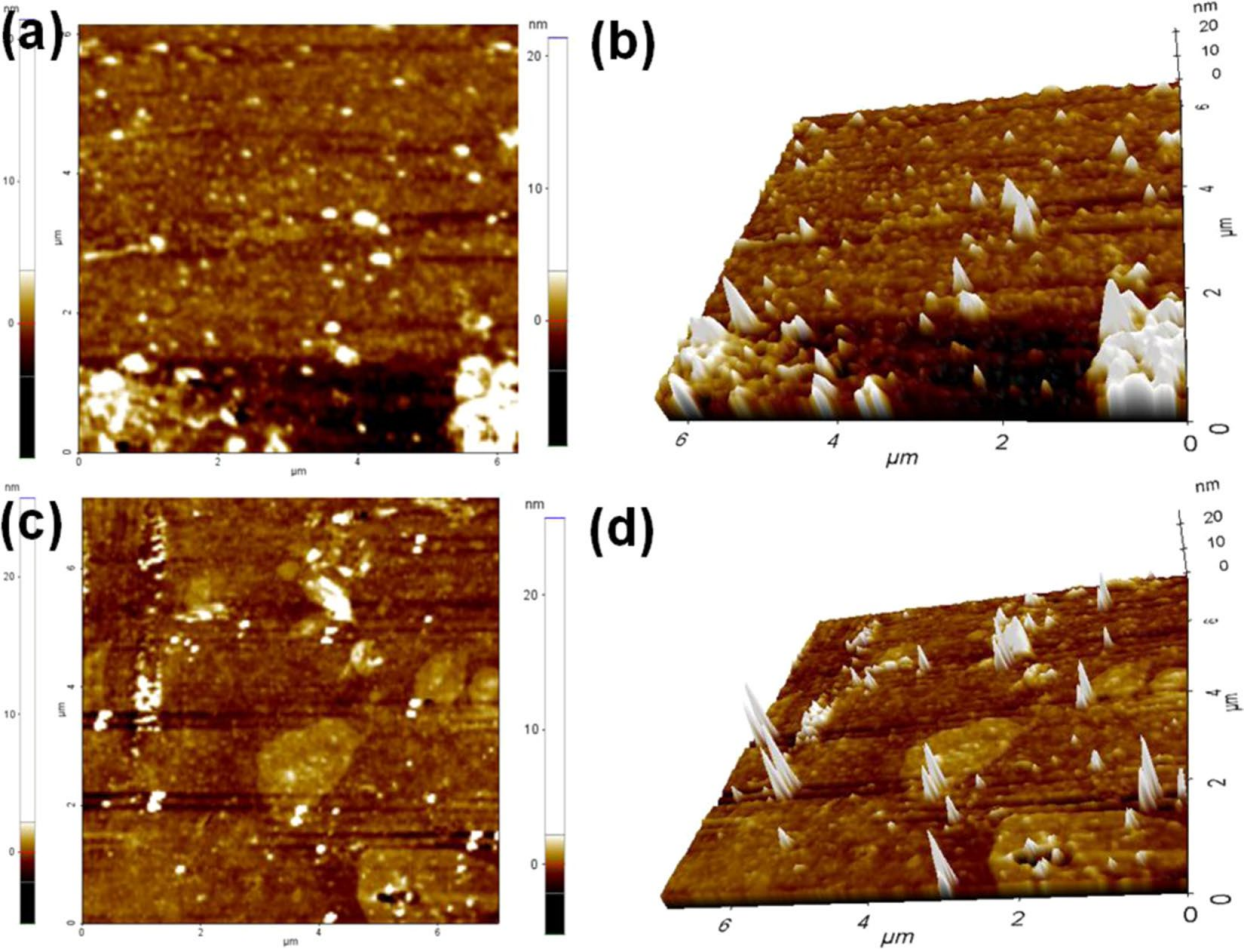
1D and 3D AFM images of (**a**,**b**) CuNi/GO-I and (**c**,**d**) CuNi/GO-II.

**Figure 5:**
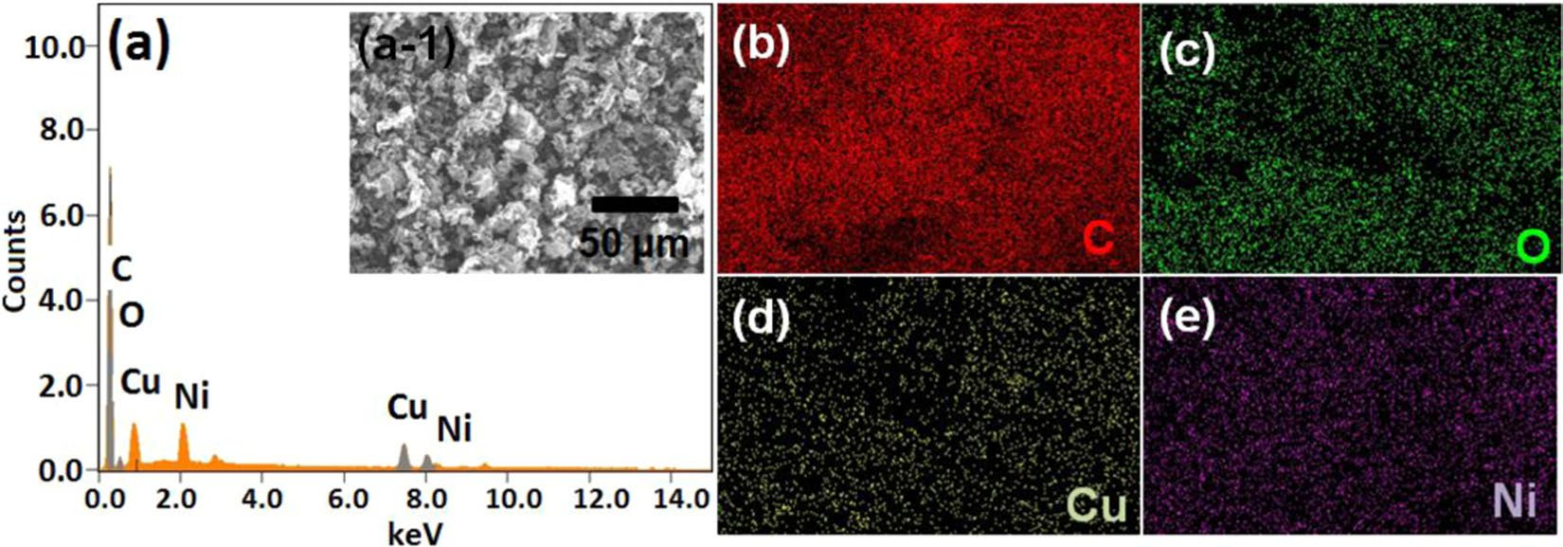
(**a**) EDS spectrum of CuNi/GO-I (the inset shows the corresponding SEM image), and (**b**–**e**) the corresponding elemental mappings of C, O, Cu and Ni for CuNi/GO-I.

To further AFM images taken for the CuNi/GO-I and CuNi/GO-II (Fig. 4). Alike HRTEM results, both one dimensional (1D) and their corresponding three-dimensional (3D) projections confirm the attachment of CuNi-oxide nanoparticles on the surface of GO. Uniform dispersion of both big and small-size CuNi-nanoparticles on GO was confirmed by the AFM images of CuNi/GO-I (Fig. 4(a,b)). AFM images of CuNi/GO-II confirmed the presence of CuNi-nanoparticles of about 12 nm on GO (Fig. 4(c,d)). AFM 3D surface profiles were captured to determine the surface roughnesses (Rq) of fresh GO, CuNi/GO-I, and CuNi/GO-II. The Rq values of GO was 93.1 nm, whereas, after metal decoration, the Rq values found to be significantly decreased (25.3 and 33.2 nm for CuNi/GO-I and CuNi/GO-II, respectively). This phenomenon is due to the decoration of CuNi-nanoparticles with GO. The AFM results agree well with the HRTEM results. To further SEM image and its corresponding EDS and elemental mapping of C, O, Cu and Ni were taken for CuNi/GO-I (Fig. 5). The content of Cu and Ni was determined to be 3.6 and 3.8 wt% respectively. Alike, the 90.8 and 1.8 wt% were calculated for C and O respectively. Moreover, the elemental mapping of Cu and Ni showed the nanoparticles homogeneously dispersed on the GO surface. No elements other than C, O, Ni and Cu were detected for CuNi/GO-I, indicating high purity of the samples. Factual loading of Cu and Ni in CuNi/GO nanocomposites was further verified by ICP-MS analysis and found that the results agree well with the EDS values.

**Figure 6:**
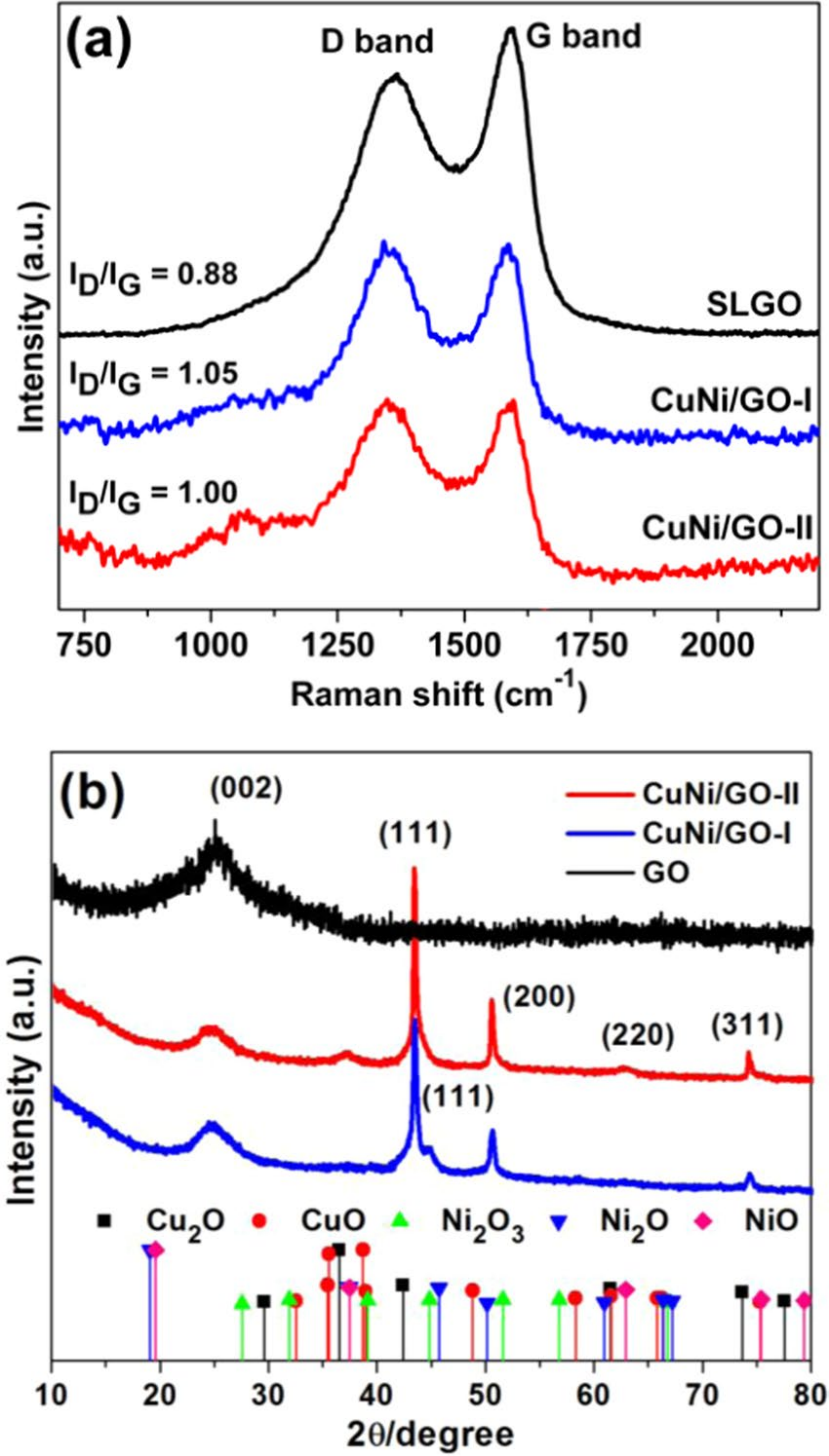
(**a**) Raman spectra and (**b**) XRD patterns of GO, CuNi/GO-I and CuNi/GO-II.

Raman spectra of fresh GO, CuNi/GO-I and CuNi/GO-II were recorded (Fig. 6a). Characteristic D and G band were noticed for all the samples. The D band at ~1340 cm^−1^ represents the presence of defect sites in GO and the G band line at ~1570 cm-1 is related to the relative degree of graphitization^27^. In order to understand the interaction of CuNi-oxide nanoparticles with GO surface, I_D_/I_G_ ratio was calculated for GO, CuNi/GO-I and CuNi/GO-II. The I_D_/I_G_ ratio of GO was 0.88, whereas, the CuNi/GO-I and CuNi/GO-II showed slightly higher values of 1.05 and 1.00, respectively. This increase in the I_D_/I_G_ values is due to the formation of more defects in GO. The creation of more defects is mainly due to the strong attachment of CuNi-oxide nanoparticles with carbon matrix of the GO. To further X-ray diffraction patterns were also recorded for fresh GO, CuNi/GO-I and CuNi/GO-II (Fig. 6b). XRD patterns of all the three samples showed a broad peak at ~25° corresponds to (002) plane of hexagonal graphite structure. In comparison to GO, four new intense peaks at 2θ = ~43.3°, 44.7°, 50.4° 62.5° and 74.1° were associated with (111), (111), (200), (220) and (311) planes for CuNi/GO-I and CuNi/GO-II. The peaks were not consistent with either a pure Cu or Ni phase (standard sites of Cu (JCPDS no. 04-0836) and Ni (JCPDS no. 04-0850)) which indicating the well-alloyed Cu-Ni oxide forms (Cu_2_O-NiO, CuO-NiO, or Cu_2_O-Ni_2_O_3_)^27,28^. Moreover, the peak nature such as intensity and broadness confirm that the CuNi-oxide nanoparticles are very small in size and nanocrystalline in nature^29^.

**Figure 7:**
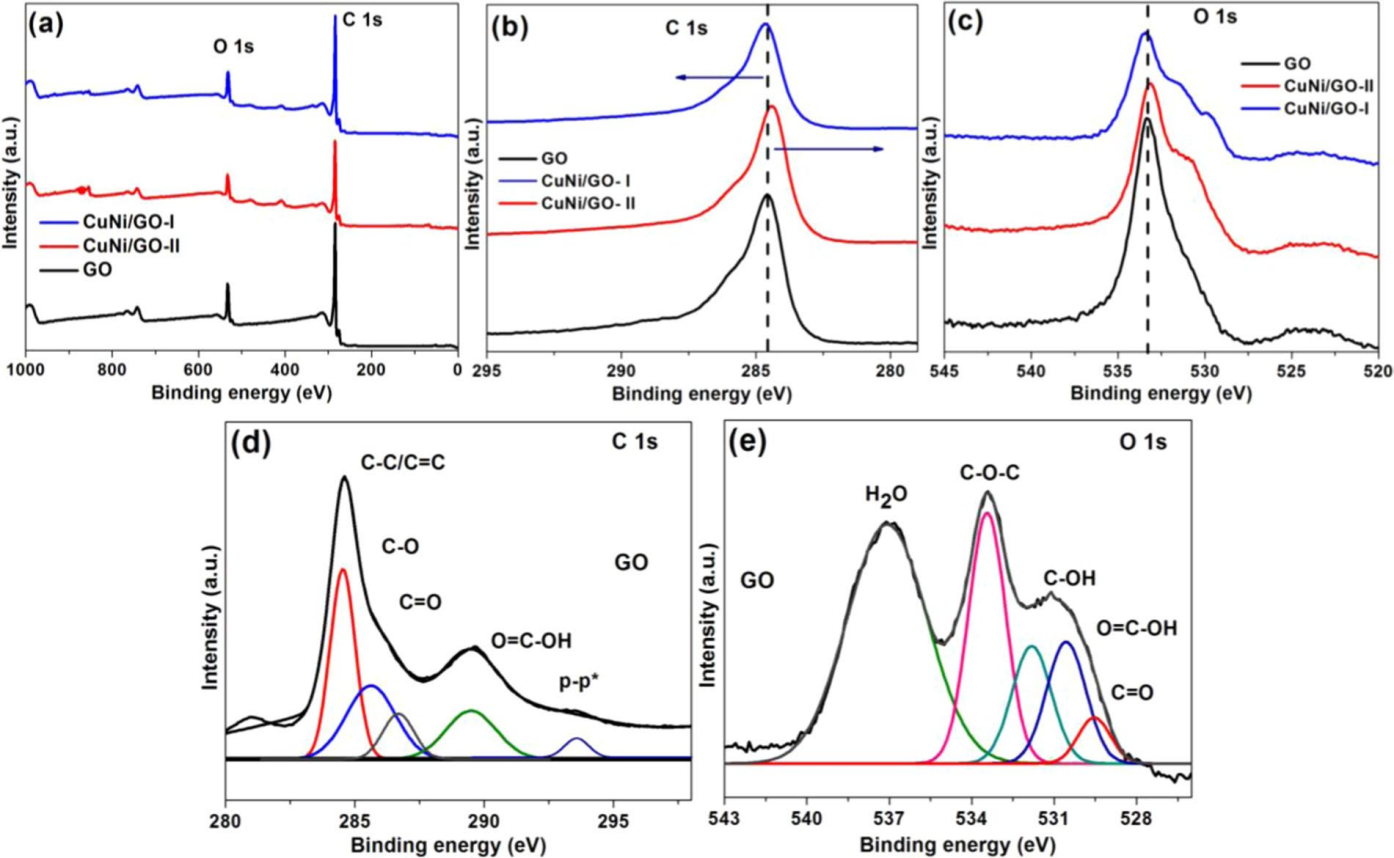
(**a**) XPS survey spectra, (**b**) C 1s XPS peaks and (**c**) O 1 s XPS peaks of GO, CuNi/GO-I and CuNi/GO-II. Deconvoluted (**d**) C 1 s and (**e**) O 1 s XPS peaks of fresh GO.

Figure 7(a–c) shows the XPS spectra of fresh GO, CuNi/GO-I and CuNi/GO-II. At first, the chemical composition of fresh GO was investigated. Two dominate peaks C 1s and O 1s were seen at BE ~285.1 and 533.4 eV, respectively (Fig. 7(b,c)). To confirm the surface functional groups of GO, peak fitting was performed on C 1s and O 1s spectra using a Gaussian–Lorentzian peak shape. Figure 7(d,e) showed the deconvolated C 1s and O 1s spectra of fresh GO. As seen, the deconvolation of C 1s and O 1s spectra resulted in four clear peaks (C 1s − 284.7, 286.8, 289.5, and 293.7 eV; O 1s − 529.5, 530.7, 532.3 and 533.4 eV) which confirm the presence of carbonyl (C = O), carboxylic (−COOH), hydroxyl (C−OH) and ether (−C−O−C−), in GO surface^30^. Moreover, the p-p* shakeup satellite peak at ~293.5 eV confirms the single or few layers of the GO^17^. These functional groups are the key factors for obtaining the excellent morphology of the CuNi/GO-I and CuNi/GO-II. In fact, the presence of oxygen function groups could assist the GO for a better processability by improving its wetness. Moreover, these function groups can be an effective anchoring sites for the CuNi-oxide nanoparticles^31^. Hence, the oxygen-rich surface of GO was chosen for the mechanochemical preparation of CuNi/GO catalysts.

**Figure 8:**
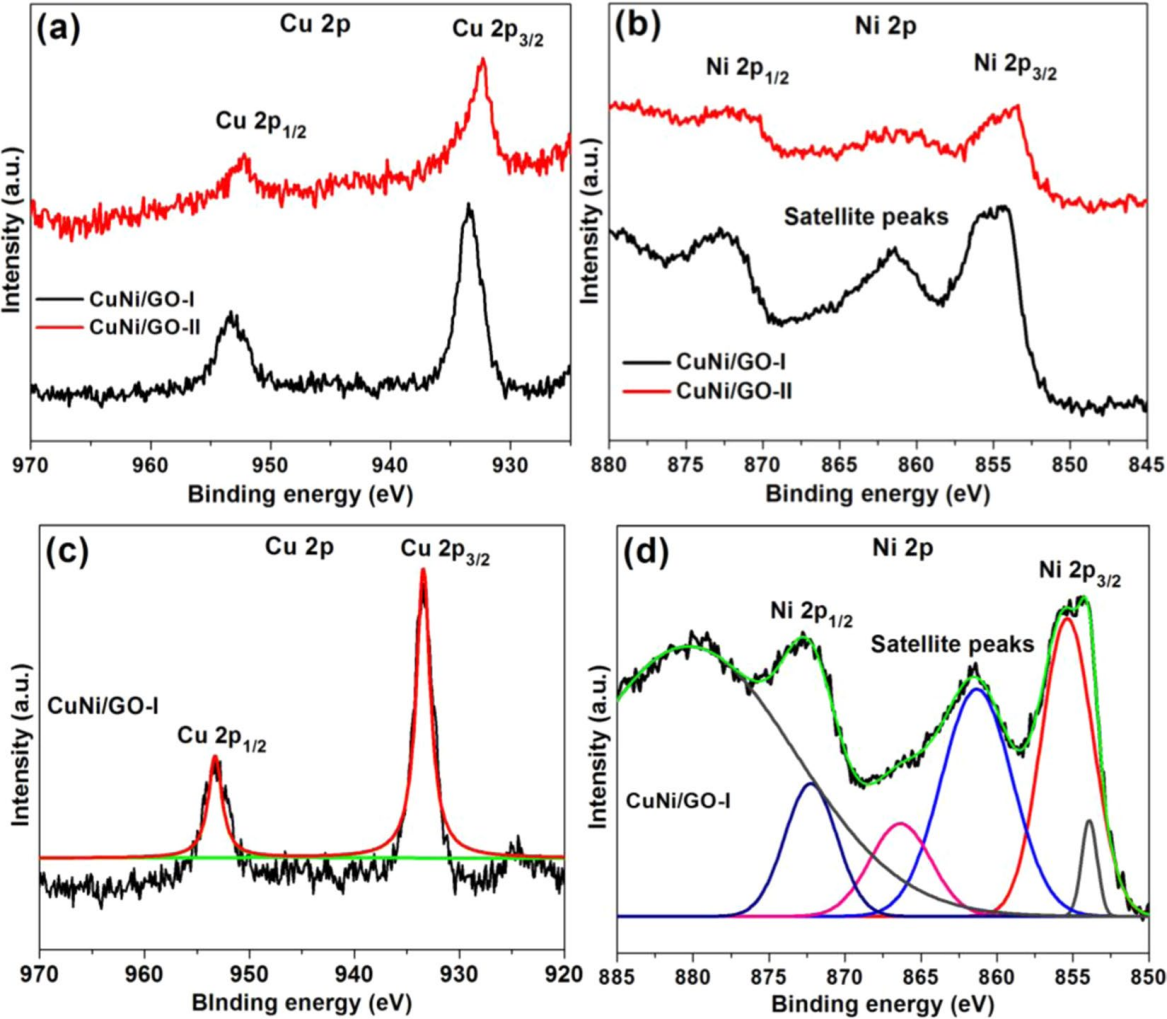
(**a**) XPS Cu 2p and (**b**) XPS Ni 2p peaks of CuNi/GO-I and CuNi/GO-II. Deconvoluted (**c**) Cu 2p and (**d**) Ni 2p peaks of CuNi/GO-I.

To further the oxidation states of Cu-Ni in CuNi/GO-I and CuNi/GO-II were investigated by XPS analysis (Fig. 8(a,b)). Alike fresh GO, both the CuNi/GO-I and CuNi/GO-II showed clear C 1s and O 1s peaks. In addition to the strong O 1s peak at 533.2 eV, two shoulder peaks at lower energy were observed which may be due to the chemisorbed or dissociated oxygen, or OH species on the surface of CuNi/GO catalysts^32^. In addition, new peaks in the Cu 2p and Ni 2p regions were observed, indicating the presence of Cu, Ni, C and O elements. To further understand the chemical state of metals, peak fitting was performed on Cu 2p and Ni 2p spectra of CuNi/GO-I (Fig. 8(c,d)). The Cu 2p XPS spectrum of CuNi/GO-I showed two dominant peaks centered at approximately 933.9 and 953.5 eV were attributed to Cu 2p_3/2_ and Cu 2p_1/2_ of monovalent Cu + (Cu_2_O), respectively^33^. Moreover, the no shake-up peak at 940~945 eV corresponded to Cu^2+^ (CuO) presented. The absence of CuO might be due to the reduction of CuO to Cu_2_O by the electron transfer from GO to CuO^34^. The peaks centered at 855.1 and 872.8 eV correspond to Ni 2p_3/2_ and Ni 2p_1/2_ spin-orbit peaks of NiO, respectively. Moreover, satellite peak at 861.5 eV (Ni 2p_3/2_ peak) were attributed to the shakeup process of NiO structure^35^. The observed peak broadness at around 858 eV clearly shows the presence of mixed Ni^2+^/Ni^3+^ phase (Ni_2_O_3_ and NiO) (Fig. 8(d)). Overall, the XRD, XPS and SAED results confirm that the bimetallic CuNi-oxide supported on GO surface were mainly well-alloyed by two or more components of CuO, Cu_2_O, NiO or Ni_2_O_3_ (most probably Cu_2_O-NiO).

**Figure 9:**
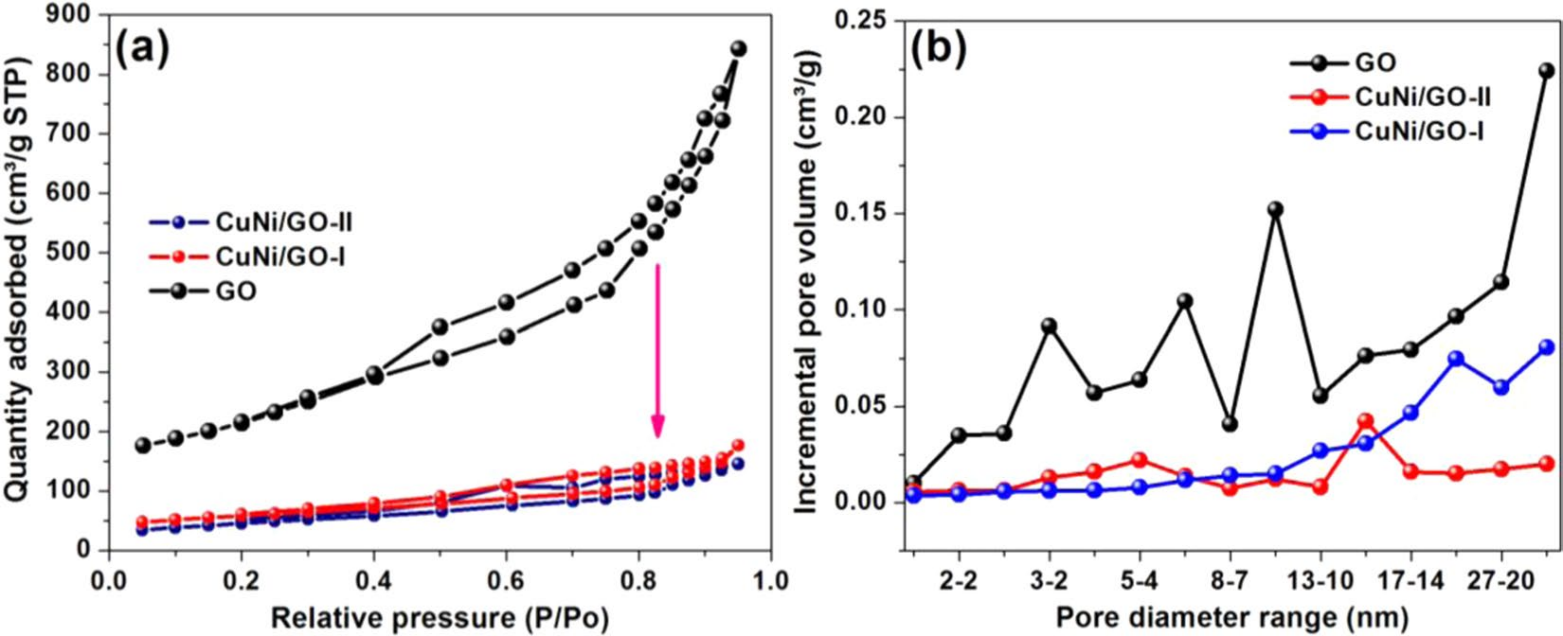
(**a**) Typical N_2_ adsorption/desorption isotherm curves of BET isotherm and (**b**) pore diameter distribution of GO, CuNi/GO-1 and CuNi/GO-II.

The N_2_ adsorption−desorption analysis was carried out for the GO, CuNi/GO-1 and CuNi/GO-II (Fig. 9). The adsorption−desorption isotherm of all the three samples exhibits a reversible type-IV adsorption isotherm, indicating the presence of micro- and macro-pores. The specific surface areas, pore volume and pore size of fresh GO was determined to be 767.3 m^2^/g, 1.29 and 7.18 nm respectively. Alike, the CuNi/GO-1 and CuNi/GO-II showed BET surface area of 165 and 151 m^2^/g, respectively. The pore volume and pore size of CuNi/GO-I (0.2252 and 5.2 nm) and CuNi/GO-II (0.2131 and 4.9 nm) were also found to be good. In comparison to the fresh GO, the surface area of CuNi/GO catalysts demonstrated low BET surface area. This is may be due to the fact that the CuNi-oxide nanoparticles were effectively occupied the pores of GO^36^. The better surface area of CuNi/GO-I (165 m^2^/g) compared to CuNi/GO-II is mainly due to the small size of the CuNi-oxide nanoparticles. Based on the BET and HRTEM analysis, it was concluded that the face-to-face aggregation of graphene layers was prevented by the decoration of CuNi-oxide nanoparticles.

Among the transition metal, Cu and Ni are readily available and less-expensive when compare to noble metal salts. As catalysts, bimetallic Cu-Ni alloys have played tremendous role in heterogeneous catalysis^24–26^. Therefore, in the present study, cost-effective GO-supported CuNi-oxide bimetallic catalyst was developed as alternate choice to the existing noble metals based catalyst for the cyanation of aryl halides. Indeed, the performance of heterogeneous catalysts is mainly dependent on the morphology, surface area, metal–support interactions, and particle size^16,19^. Hence, we believed that the present bimetallic CuNi/GO catalyst can be effectively used as catalyst for the cyanation reaction. For comparison, the Cu-oxide/GO-I and Ni-oxide/GO-I were prepared and characterized by means of TEM, XPS and EDS (Refer Figs. S1–S7 in the Supporting Information).

### Cyanation of aryl halides with K_4_Fe(CN)_6_

After being characterized, the bimetallic CuNi/GO-I was applied for the cyanation of aryl halides with K_4_[Fe(CN)_6_]. Reaction conditions such as catalysts, amount of catalyst, base, solvent and temperature were optimized (Table 1). Cycnation reaction of 1,4-dibromobenzene to 1,4-dicyanobenzene was selected as a modal reaction for screening. As expected, the reaction was not preceded in the absence of catalyst (Table 1, entry 1). Subsequently, the amount of CuNi/GO-I catalyst was optimized (Table 1, entries 2–4). Surprisingly, a 92% of the desired product was obtained by the CuNi/GO-I catalyst (Table 1, entry 3). Different amount of the catalyst (10, 15 and 20 mg) was used, in which 15 mg of the catalyst (0.92 mol% of Cu and 0.95 mol% of Ni) was found to be efficient enough to afford the 1,4-dicyanobenzene in excellent yield (Table 1, entry 3). To find out the suitable base for the reaction, K_2_CO_3_, KOH, Na_2_CO_3_, and Et_3_N were used (Table 1, entries 3, 5, 6 and 7). The KOH and Et_3_N were found to be less efficient, whereas, the Na_2_CO_3_ afford a moderate yield of 58% (Table 1, entries 5–7). Maximum amount of 92% yield was archived when 1.2 mmol of K_2_CO_3_ was used as base (Table 1, entry 3). Similarly, solvent is also played significant role in the present catalytic system. DMSO, toluene, H_2_O and DMF were used, whereas, the reaction found to be highly effective with DMF (Table 1, entries 3, 8, 9 and 10). A trace amount of the desired product was obtained from the reaction proceed at room temperature (29 °C) (Table 1, entry 11), whereas, the reaction stirred at 100 °C afford 47% of the desired product (Table 1, entry 12). Hence, 120 °C was chosen to be an optimum temperature. To achieve the maximum conversion of reactant to product, all the reactions were stirred for 24 h under optimum conditions. It was found that the present system does not require excess amount of K_4_Fe(CN)_6_, as each mole of K_4_Fe(CN)_6_ contained six cyanide ions. Therefore, the ratio of K_4_Fe(CN)_6_ to aryl halide was 0.17:1.

**Table 1:** Standardization of reaction conditions^a^.


S. No	Catalyst, amount (mg)	Base	Solvent	Temperature (°C)	Yield (%)
1	0	K_2_CO_3_	DMF	120	Trace
2	CuNi/GO-I, 10	K_2_CO_3_	DMF	120	69
3	CuNi/GO-I, 15	K_2_CO_3_	DMF	120	92
4	CuNi/GO-I, 20	K_2_CO_3_	DMF	120	93
5	CuNi/GO-I, 15	KOH	DMF	120	43
6	CuNi/GO-I, 15	Na_2_CO_3_	DMF	120	58
7	CuNi/GO-I, 15	Et_3_N	DMF	120	12
8	CuNi/GO-I, 15	K_2_CO_3_	DMSO	120	81
9	CuNi/GO-I, 15	K_2_CO_3_	Toluene	120	21
10	CuNi/GO-I, 15	K_2_CO_3_	H_2_O	120	Trace
11	CuNi/GO-I, 15	K_2_CO_3_	DMF	27	Trace
12	CuNi/GO-I, 15	K_2_CO_3_	DMF	100	47
13	CuNi/GO-II, 15	K_2_CO_3_	DMF	100	78
14	Cu-oxide/GO-I, 15	K_2_CO_3_	DMF	100	16
15	Ni-oxide/GO-I, 15	K_2_CO_3_	DMF	100	9

^a^Reaction conditions: 1,4-Dibromobenzene (1 mmol), K_4_[Fe(CN)_6_].3H_2_0 (0.34 mmol), base (1.2 mmol), solvent (5 mL), 120 °C, 24 h.

^b^Isolated yield.

To further the CuNi/GO-II, Cu-oxide/GO-I and Ni-oxide/GO-I were also investigated for the cyanation of aryl halides under the optimized reaction conditions (Table 1, entries 13, 14 and 15). The CuNi/GO-II gave moderate yield of 78%, whereas, the mono metallic catalysts afford a very low amount of the target product, 1,4-dicyanobenzene. It clearly shows that the CuNi/GO-I is highly suitable for the cyanation reaction when compared to CuNi/GO-II, Cu-oxide/GO-I and Ni-oxide/GO-I. Overall, the results suggested that the best yields (71–95% product) could be obtained with 15 mg of CuNi/GO-I, 0.34 equiv of K_4_[Fe(CN)_6_].H_2_O, 1.2 mmol of K_2_CO_3_, in DMF after heating the reaction mixtures at 120 °C under an inert atmosphere for 24 h (Table 1, entry 3). The scope of the present CuNi/GO-I based catalytic system was extended with a wide range of substrates such as aryl iodides, aryl bromides, aryl iodides and heteroaryl compounds.

Table 1 shows the present CuNi/GO-I system activity transformed a wide range of substrates such as aryl iodides, aryl bromides, aryl iodides and heteroaryl compounds to corresponding nitriles. Under the optimized condition, the 1,4-dibromobenzen was transformed to 1,4-dicyanobenzene in an excellent 92% yield (Table 2, entry 1) whereas Pd/CuO system affords 86% of the product^12^. The TON/TOF values were calculated to be 49/2 h^−1^. Cyanation of iodobenzene yielded 95% of benzonitrile with good TON/TOF values of 50/2 h^−1^ (Table 2, entry 2). However, Cu-based homogenous system yielded 83% of benzonitrile from the cyanation of iodobenzene^37^. Similarly, 95% of 4-methoxybenzonitrile was isolated from the CuNi/GO-I catalyzed cyanation of 1-iodo-4-methoxybenzene (Table 2, entry 3). The TON/TOF value was calculated to be 50/2 h^−1^. Bromobenzene was also effectively transformed to benzonitrile in 91% yield with TON/TOF of 49/2 h^−1^ (Table 2, entry 4). Alike, heteroaryl halide, 4-bromopyridine was successfully converted to 4-cyanopyridine and gave an isolated yield of 84% with TON/TOF of 45/2 h^−1^ (Table 2, entry 5). Most of the non-noble metal based catalysts are not active towards the aryl chlorides. For instance, Zhu *et al*.^38^, showed non-activated C-Cl bonds are inert when the reaction was stirred with CuI as catalyst (Reaction condition: aryl chloride, oxylene, Na_2_CO_3_, CuI/additive, acetone cyanohydrin in oxylene, 26 h reaction time, 150 °C). Heterogeneous Cu/C catalytic system is also inactive towards the cyanation of aryl bromides and chlorides^13^. Similarly, Pd/CuO nanocatalyst was also found to be less efficient toward the cyanation of aryl chlorides with K_4_Fe(CN)_6_. Surprisingly, cyanation reaction of chlorobenzene was well carried by the present CuNi/GO-I system (Table 2, entry 6). A better 71% yield of cyanobenzene was obtained from the cycnation reaction of chlorobenzene (Table 2, entry 6) whereas PdNCs/C@Fe_3_O_4_ system gave 23% of the product even after the reaction was stirred at 120 °C for 32 h^11^. It was found that the present CuNi/GO-I system is also applicable for the cyanation of substituted aryl halides. The present CuNi/GO-I transformed the *para* substituted NO_2_- and CHO- nitrobenzene to corresponding aromatic nitriles in excellent yields with good TON/TOF values of 50–47/2 h^−1^ (Table 2, entries 7 and 8). The isolated yields of 4-nitrobenzonitrile and 4-formylbenzonitrile were calculated to be 93 and 88% respectively.

**Table 2:** Scope extension^a^.

S. No	Aryl halide	Product	Yield (%)^b^	TON/TOF h^−1^
1			92	49/2
2			95	50/2
3			95	50/2
4			91	49/2
5			84	45/2
6			71	38/2
7			93	50/2
8			88	47/2
9			81	43/2
10			85	46/2
11			89	48/2
12			91	49/2

^a^Reaction conditions: 1,4-Dibromobenzene (1 mmol), K_4_[Fe(CN)_6_].3H_2_0 (0.34 mmol), CuNi/GO-I (15 mg, 0.92 mol% of Cu and 0.95 mol% of Ni), base (1.2 mmol), solvent (5 mL), 120 °C, 24 h.

^b^Isolated yield.

^c^TON/TOF [TON = the amount of product (mol)/the amount of active sites; TOF = TON/time (h)].

In general, conversion of aromatic polyhalides, like tribromobenzene, is often hard under normal reaction conditions, especially, by using non-noble metal catalysts. To our delight, the present CuNi/GO-I was found to be highly active for the aromatic polyhalides. The 1,3,5-tribromobenzene was successfully converted to benzene-1,3,5-tricarbonitrile and the yield was calculated to be 81% and the TON/TOF values were 43/2 h^−1^. The activity of the present CuNi/GO-I can be compared with Pd@CC1^r^/Pd@CC2^r^-catalytic system^10^. Alike the cyanation of 1,4-dibromobenzene, the CuNi/GO-I transformed 4-bromobenzonitrile to 1,4-dicyanobenzene in an good yield of 85% with TON/TOF of 46/2 h^−1^ (Table 2, entry 10). Under the optimum conditions, the CuNi/GO-I converts 1-chloro-4-iodobenzene and 1-bromo-4-chlorobenzene to corresponding nitrile, 4-chlorobenzonitrile, in good yields (Table 2, entries 11 and 12). The results confirmed that the present non-noble metal catalyst is highly efficient and the reactions are able to tolerate a wide range of substrates. Based on the results, we concluded that the catalytic activity of the CuNi/GO-I is mainly due to its unique morphology, very small size of CuNi oxide nanoparticels, well composition of Cu and Ni (high synergy), high surface area, fine dispersion in reaction medium, and the presence of high defect sites. To the best of our knowledge this is this first efficient bimetallic heterogeneous catalyst reported for the cyanation of aryl halides with K_4_[Fe(CN)_6_].3H_2_0.

**Figure 10:**
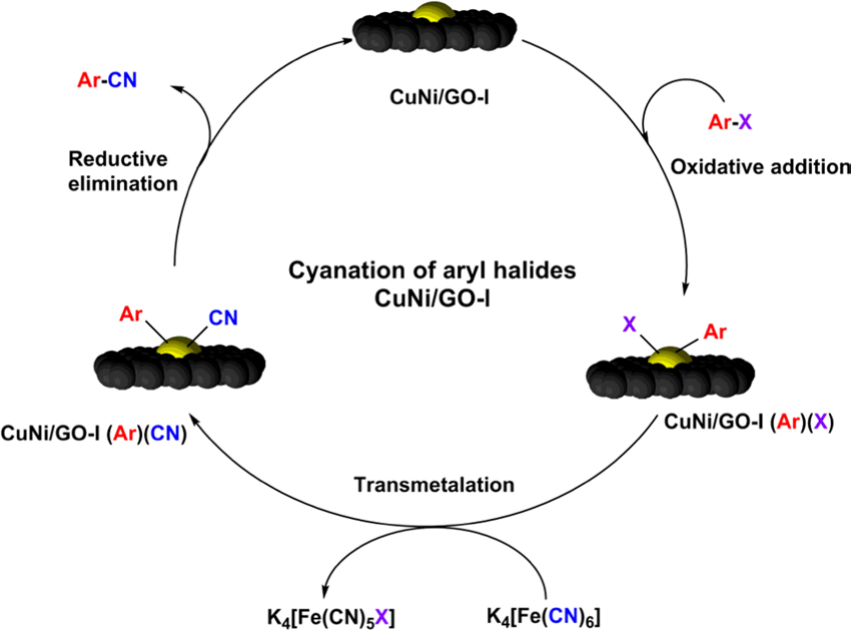
Proposed mechanism for the CuNi/GO-I catalyzed cyanation of aryl halides. (ACD Lab (https://www.acdlabs.com/) and ChemDraw Professional 16.0 (http://www.cambridgesoft.com/Ensemble_for_Chemistry/details/Default.aspx?fid=14&pid=736&l=en) software were used to draw the image).

Possible reaction mechanism was proposed for the CuNi/GO-I catalyzed cyanation of aryl halides (Fig. 10). At first, stirring of aryl halide and CuNi/GO-I leads to the adsorption of aryl halide species on CuNi-oxide nanoparticles surface *via* oxidative addition. Subsequently, transmetalation takes place between K_4_[Fe(CN)_6_] and aryl halide species adsorbed CuNi site and forms complex CuNi/GO-I(Ar)(X). Finally, the reductive elimination takes place in CuNi/GO-I(Ar)(CN) to give the final product. The CuNi/GO-I catalyst was regenerated for the further cyanation reaction.

#### Reduction of 4-nitrophenol

**Figure 11:**
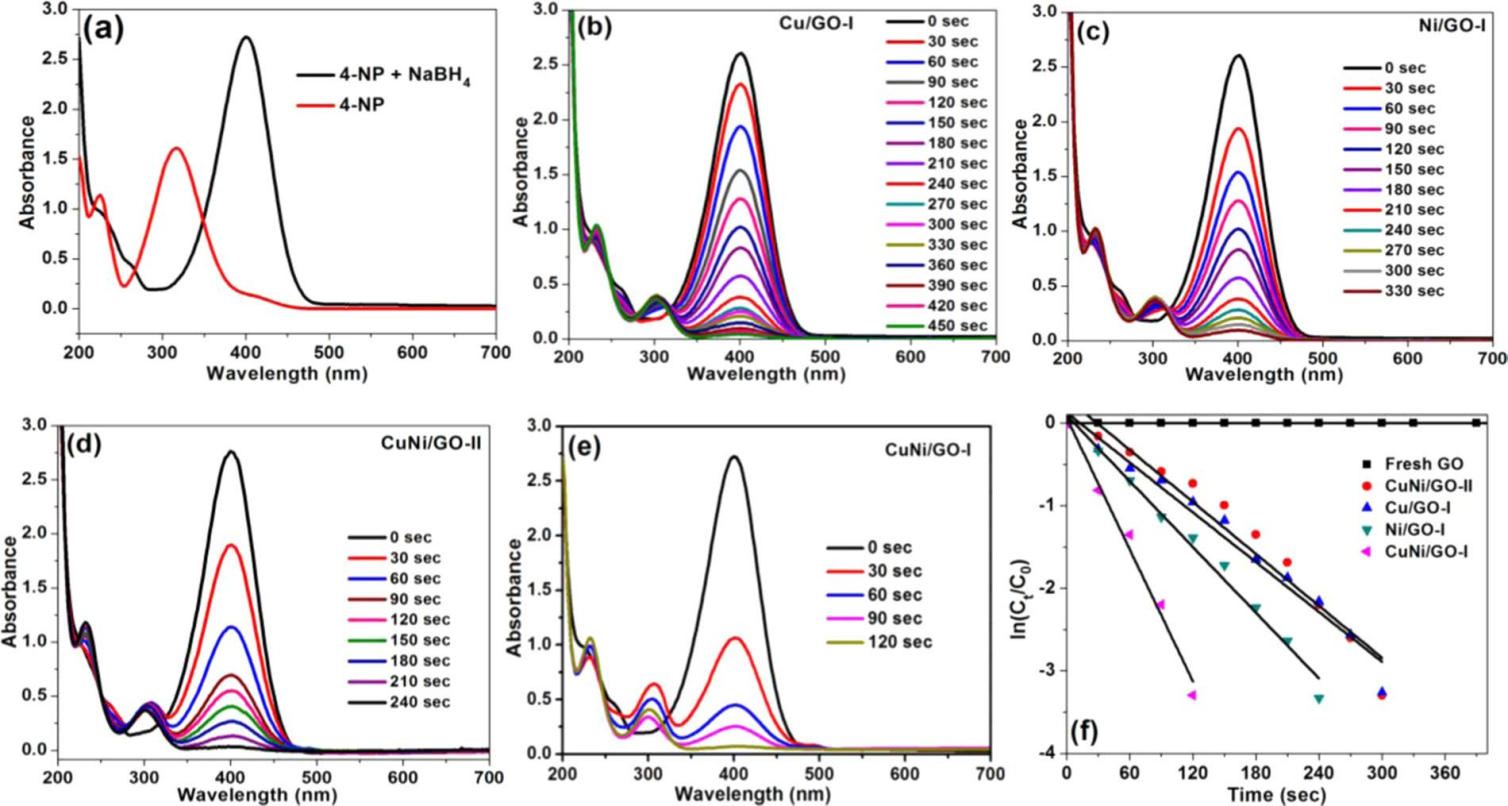
(**a**) UV-vis spectra of 4-nitrophenol before and after adding NaBH_4_ solution. UV-vis spectra for the reduction of 4-nitrophenol by using (**b**) Cu-oxide/GO-I, (**c**) Ni-oxide/GO-I, (**d**) CuNi/GO-II, and (**e**) CuNi/GO-I. (**f**) Plots of ln[Ct/C0] *versus* reaction time for reduction of 4-nitrophenol with NaBH_4_ over Cu-oxide/GO-I, Ni-oxide/GO-I, CuNi/GO-II, and CuNi/GO-I.

Inspired by the results obtained from the cyanation reaction, the present mono and bimetallic nanocatalysts (Cu-oxide/GO-I, Ni-oxide/GO-I, CuNi/GO-I and CuNi/GO-I) were tested for the reduction of 4-nitrophenol in the presence of NaBH_4_. To our delight, the versatility of CuNi/GO-I was confirmed by its superior catalytic activity in the reduction of 4-nitrophenol to 4-aminophenol. At first, reaction condition was optimized. As a result, 80 μL of 0.01 M 4-nitrophenol, and 4 mL of 0.015 M aqueous NaBH_4_ were fixed to be the optimum one. As expected, 0% conversion of 4-nitrophenol was noticed in the absence of catalyst. The catalyst amount of 0.1 mg was found to be enough. Figure 11(a) shows the UV-vis spectra of 4-nitrophenol before and after adding NaBH_4_ solution. The pure 4-nitrophenol in water showed absorption band at 317 nm, whereas, upon the addition of NaBH_4_, the band red-shifted to 400 nm, indicating the formation of 4-nitrophenolate ion. The peak at 400 nm was observed to be rapidly decreased when a small amount of catalyst was introduced into the reaction mixture. Surprisingly, all the present mono and bimetallic catalysts (Cu-oxide/GO-I, Ni-oxide/GO-I, CuNi/GO-I and CuNi/GO-II) were found to be efficient for the reduction of 4-nitrophenol as it afford 100% of the catalytic product, 4-aminophenol (Fig. 11(b–e)). Among them the CuNi/GO-I demonstrated better performance over Cu-oxide/GO-I, Ni-oxide/GO-I, CuNi/GO-II. The CuNi/GO-I reduced 4-nitrophenol in just 120 sec whereas CuNi/GO-II required 240 sec. Similarly, 450 and 330 sec were required for the mono metallic catalysts Cu-oxide/GO-I and Ni-oxide/GO-I respectively (Fig. 11(b–e)).

Using the time-dependent UV-Vis spectra, reaction kinetics on the reduction of 4-nitrophenol by Cu-oxide/GO-I, Ni-oxide/GO-I, CuNi/GO-I and CuNi/GO-II was studied (Fig. 11(f)). The liner fit between ln(*C*_*t*_/*C*_0_) and time confirm the reduction reaction follows pseudo-first-order reaction kinetics (Fig. 11(f)). The kinetic reaction rate constant (k_app_) values were calculated from the slope of the liner fit. The values acknowledge the superior activity of the present catalyst. The k_app_ values of 5.60, 5.91, 7.92 and 18.2 × 10^−3^ s^−1^ were calculated for the 4-nitrophenol reduction reaction catalyzed by 0.1 mg of Cu/GO-I, Ni/GO-I, CuNi/GO-II, and CuNi/GO-I, respectively. The better k_app_ value of CuNi/GO-I is due to its smaller particles size and high synergetic effect. The catalytic performance of the present catalysts can be compared over perilously reported heterogeneous catalysts (Table 3). For example, bimetallic CuNi nanocrystals used for the reduction 4-nitrophenol and the k_app_ value was calculated to be of 9.7 × 10^−3^ s^−1^ whereas the present CuNi/GO-I system reached a higher k_app_ value of 18.2 × 10^−3^ s^−1^ ^39^. Alike, k_app_ values of 0.89 min^−1^ and 0.030 s^−1^ g^−1^ L were reported for the 4-nitrophenol reduction catalyzed by hollow CuNi alloy nanoparticles on reduced graphene oxide nanosheets (RGO–CuNi) and Ni-supported Cu-MOF (Ni/NPC-900)^40,41^. The results confirmed that the present CuNi/GO-I is better for the reduction 4-nitrophenol when compare to other bimetallic Cu-Ni nanocatalysts. Based on the results, mechanism has been proposed for the CuNi/GO-I catalyzed reduction reaction. At first, stirring the mixture of CuNi/GO-I, NaBH_4_ and 4-nitrophenol, leads to the adsorption of nitrophenolate on to the CuNi-nanoparticles of catalyst and produces active hydrogen atoms on the catalyst surface by BH^4−^. Subsequently, the formed hydrogen atom reduces 4-nitrophenol to yield 4-aminophenol. In the present case, the graphene support could have assisted as an excellent interfacial electron relay medium for the rapid transfer of electron sources from BH_4_^−^ to the CuNi-oxide surface. This could be further enhanced by the well-alloyed Cu-Ni oxide nanoparticles supported on GO.

**Table 3:** Comparison of present CuNi/GO-I nanocomposite over other heterogeneous catalysts.

S. No	Catalyst (amount used, mg)	k_app_ (× 10^−3^ s^−1^)	k̍ (× 10^−3^ mg^−1^ s^−1^)	TOF (s^−1^)	References
1	CuNi/GO-I (0.1)	18.2	182	107	This work
2	CuNi/GO-II (0.1)	7.92	79.2	98.9	This work
3	15 wt.% Ni/SNTs	20.0	44.0	—	^42^
4	Ni/GO-2 (0.75)	35.4	47.2	25.33	^43^
5	Cu/perlite (10)	27.0	2.7	—	^44^
6	Ni/MC-750 (3)	6.26	20.9	1.44	^45^
7	CuONPs@CNs	6.9	—	0.25	^46^
8	PdNiP/RGO	23.51	7.7	—	^47^
9	Pt–Ni/RGO (3)	3.70	1.23	110.9	^48^
10	CuO–rGO (10)	13.95	1.40	—	^49^
11	Ni/SNTs (4)	2.7	2.6	—	^42^
12	Cu/C	0.33	0.16	—	^50^
13	Cu@Ni-NWs/G (2)	6.0	3.0	—	^51^
14	Ni/MC-950	2.4	3.4	1.43	^45^
15	Ni-Pd/NrGO	17.0	—	—	^52^

TOF, s^−1^: (turnover frequency) moles of 4-NP converted per mole surface of CuNi per second.

#### Synergistic effect

Catalytic enhancement of bimetallic systems can be explained by synergistic effect^27^. The synergy with bimetallic catalysts is directly related to the effective combination of two different metals. The yields obtained from cyanation of 1,4-biromobenzene by mono and bimetallic catalysts were taken to study the synergistic effect. The mono metallic catalysts, Cu-oxide/GO-I and Ni-oxide/GO-I, afforded the target product in poor yield of 16 and 9%, respectively, where as the bimetallic system gave 92%. Synergistic effect was calculated by using the formula; synergy = (% yield) of bimetallic catalyst/(% yield) of monometallic catalysts. To our delight, the present CuNi/GO-I demonstrated about 3.7 fold of enhancement when compared to that of the mono metallic Ni-oxide/GO-I and Cu-oxide/GO-I systems. Similarly, the synergy of mono and bimetallic catalysts towards the reduction of 4-nitrophenol was also investigate. The 48 and 42% conversion of 4-nitrophenol was respectively achieved by Cu-oxide/GO-I and Ni-oxide/GO-I, where as the bimetallic CuNi/GO-I showed 100% conversion. About 1.2 fold enhancement of the yields with the CuNi/GO-I was calculated. As confirmed by HR-TEM, the well combination of Cu and Ni is the key factor for the significant catalytic enhancement of the bimetallic system. In addition, other main factors such as unique morphology, small size of CuNi oxide nanoparticels, high surface area, and the presence of high defect sites are also the reasons for the excellent performance. The catalytic performance of CuNi/GO-II found to be slightly lower when compared to CuNi/GO-I which may be due to difference in the size of CuNi-oxide nanoparticles.

#### Heterogeneity, reusability and stability

**Figure 12:**
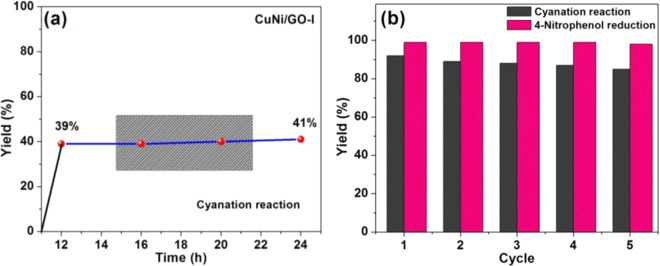
(**a**) Heterogeneity of CuNi/GO-1 in cyanation of aryl halides, and (**b**) Reusability of CuNi/GO-I in cyanation of aryl halides and reduction of 4-nitrophenol.

Very important natures such as heterogeneity, reusability and stability were tested for the present bimetallic of CuNi/GO-I catalyst (Fig. 12). Heterogeneous nature of the CuNi/GO-1 was confirmed by a hot filtration test. In brief, the cyanation of 1,4-dibromobenzene under optimized reaction conditions was carried out for 12 h. Subsequently, the catalyst was removed from the reaction mixture and then the reaction was continued stirring for 12 h. The catalyst gave 39% of the product after stirring for 12 h, whereas, after removing the catalyst, no significant increase in the yield was noticed (Fig. 12(a)). It confirmed that the CuNi/GO-I catalyst is highly heterogeneous and there is no leaching of metal catalyst during the reaction.

To further the reusability and stability of the catalyst was studied. Surprisingly, the present catalyst is highly reusable and stable. Prior to reuse, the CuNi/GO-I was washed well with diethyl ether and dried at 120 °C. Figure 12(b) shows the reusability of CuNi/GO-I in the cyanation of 1,4-dibromobenzene and reduction of 4-nitrophenol. After 5^th^ cycle, the CuNi/GO-I system afford 82% of the 1,4-dicyanobenzene. Surprisingly, 100% conversion of 4-nitrophenol was achieved by CuNi/GO-I even after 5^th^ use (Fig. 12b). The used catalyst was characterized by TEM and found that the morphology of the CuNi/GO-I is almost similar to that of the fresh catalyst. The results confirmed the highly heterogeneous, reusable, stable nature of the present CuNi/GO-I catalyst.

## Conclusions

Highly efficient bimetallic CuNi/GO-I catalyst was prepared by a very simple and feasible mechanochemical synthesis method for the cyanation of aryl halides. To the best of our knowledge, this is the first efficient bimetallic heterogeneous catalyst (noble metal free) developed for the cyanation of aryl halides with K_4_[Fe(CN)_6_].3H_2_O. A wide range of substrates such as aryl iodides, aryl bromides, aryl iodides and heteroaryl compounds (Yields: 95–71%, TON/TOF: 50–38/2 h^−1^) can be actively transformed by CuNi/GO-I. Moreover, the CuNi/GO-I demonstrated enhanced catalytic performance in the reduction of 4-nitropehnol with NaBH_4_ (k_app_ = 18.2 × 10^−3^ s^−1^ with 0.1 mg of CuNi/GO-I). The CuNi/GO-I is stable and heterogeneous in nature, and it can be reused several times without significant loss in the catalytic activity. Overall, due to high activity, reusability, versatility and cost-effectiveness, the present CuNi/GO-I would be an alternate choice for the noble-metal based catalysts to perfume the cyanation of aryl halides with K_4_[Fe(CN)_6_].3H_2_O.

## Supplementary information

Supplementary Information.
