# Supplementary material for: Sea-Island-Like Morphology of CuNi Bimetallic Nanoparticles Uniformly Anchored on Single Layer Graphene Oxide as a Highly Efficient and Noble-Metal-Free Catalyst for Cyanation of Aryl Halides
Source: Sci Rep. 2020 Jan 20;10:677. doi: 10.1038/s41598-020-57483-z (PMC6971289; doi:10.1038/s41598-020-57483-z)
Supplement: Supplementary file 1 — Supplementary Information. [file 41598_2020_57483_MOESM1_ESM.doc]

**Supporting Information**

**Sea-Island-Like Morphology of CuNi Bimetallic Nanoparticles Uniformly Anchored on Single Layer Graphene Oxide as a Highly Efficient and Noble-Metal-Free Catalyst for Cyanation of Aryl Halides**

Mayakrishnan Gopiraman,^1^ Elayappan Vijayakumar,^2^ Ick Soo Kim^3^ and Ill-Min Chung^1^,*

^1^Department of Applied Bioscience, College of Life & Environment Science, Konkuk University, 120 Neungdong-ro, Gwangjin-gu, Seoul 05029, South Korea

^2^Department of Materials Science and Technology, Korea University, Seoul- 02841, South Korea

^3^Nano Fusion Technology Research Group, Division of Frontier Fibers, Institute for Fiber Engineering (IFES), Interdisciplinary Cluster for Cutting Edge Research (ICCER), Shinshu University, Tokida 3-15-1, Ueda, Nagano Prefecture 386-8567, Japan

*Corresponding author. Tel.: +82 02 450 3730; Fax: +82 02 446 7856.

E-mail addresses: [illminchung@gmail.com](mailto:illminchung@gmail.com) (I.M. Chung).


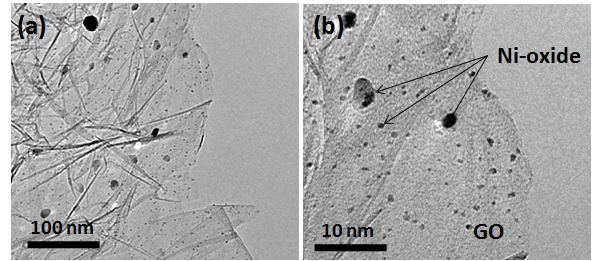


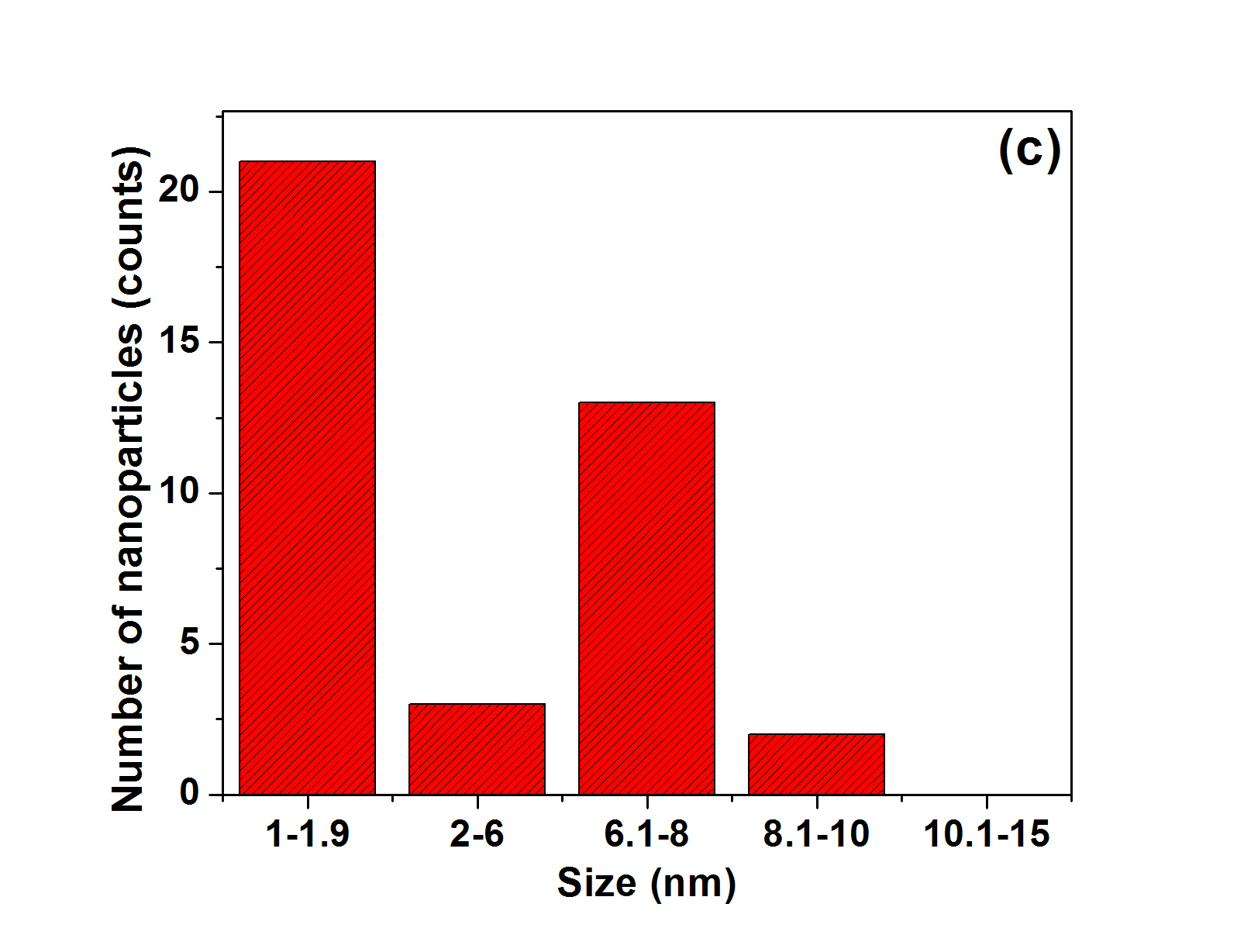


**Fig. S1**. (a and b) TEM images of Ni-oxide/GO-I. (c) Particle-size distribution histogram of Ni-oxide nanoparticles in Ni-oxide/GO-I.


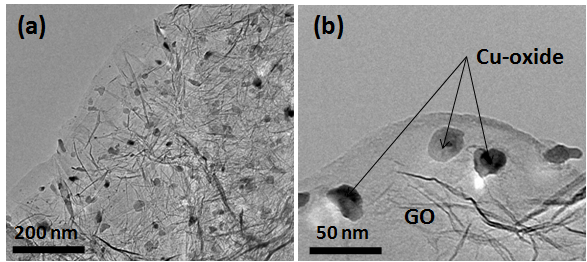


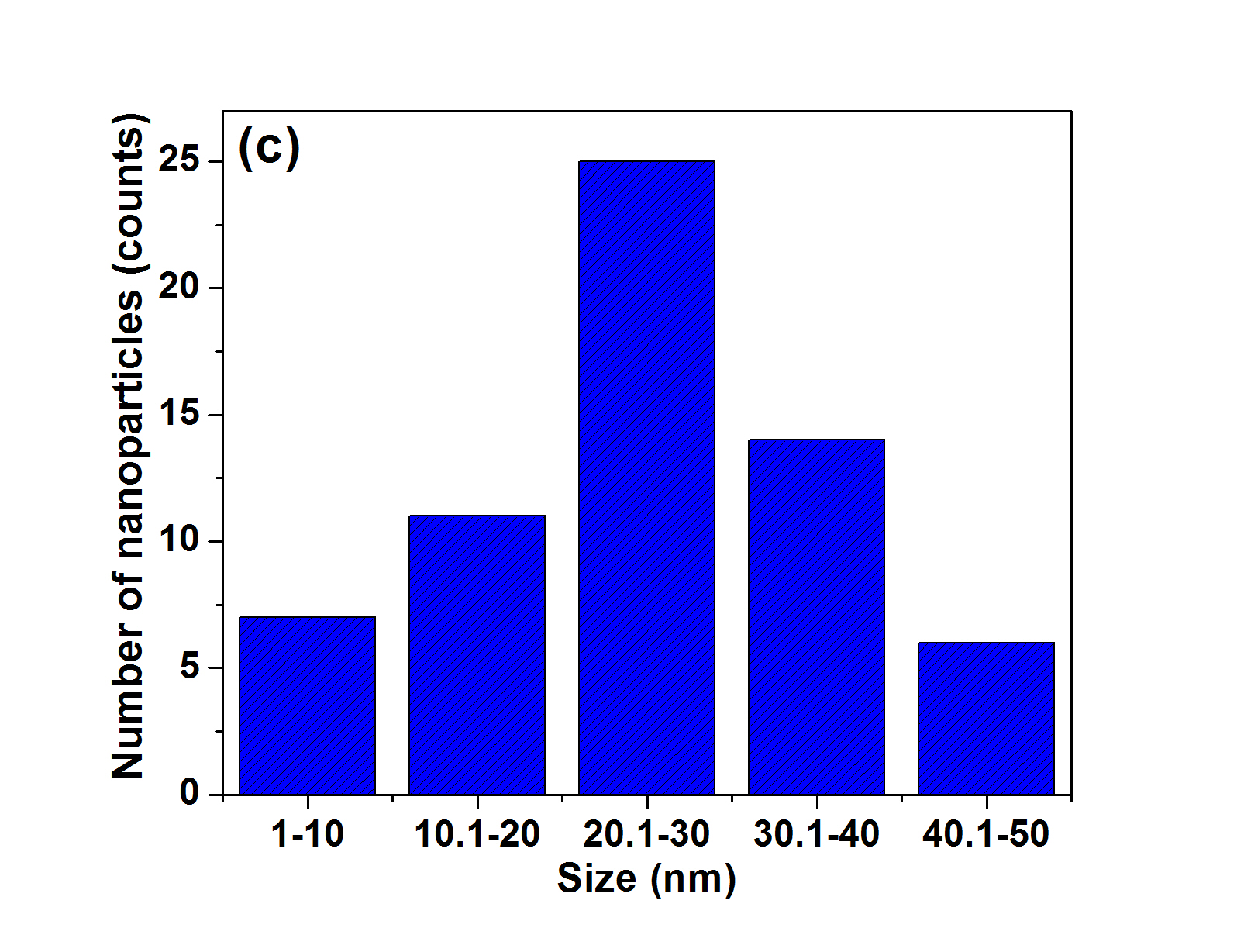


**Fig. S2**. (a and b) TEM images of Cu-oxide/GO-I. (c) Particle-size distribution histogram of Cu-oxide nanoparticles in Cu-oxide/GO-I.


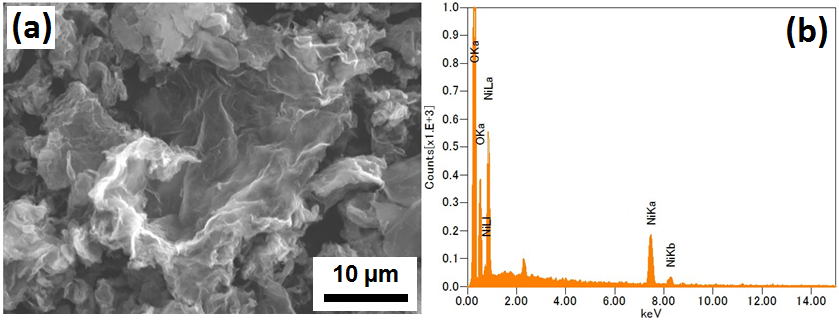


**Fig. S3**. (a) SEM image and (b) EDS spectrum of Ni-oxide/GO-I.


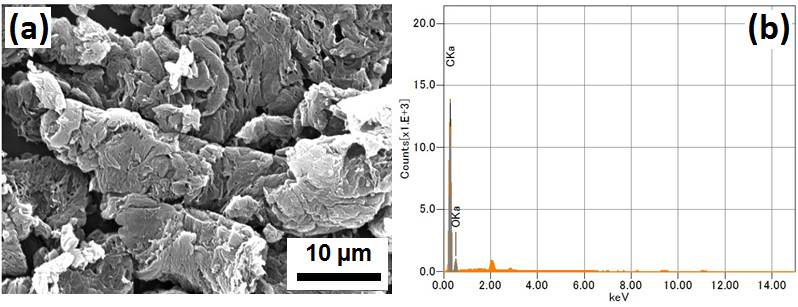


**Fig. S4**. (a) SEM image and (b) EDS spectrum of GO.


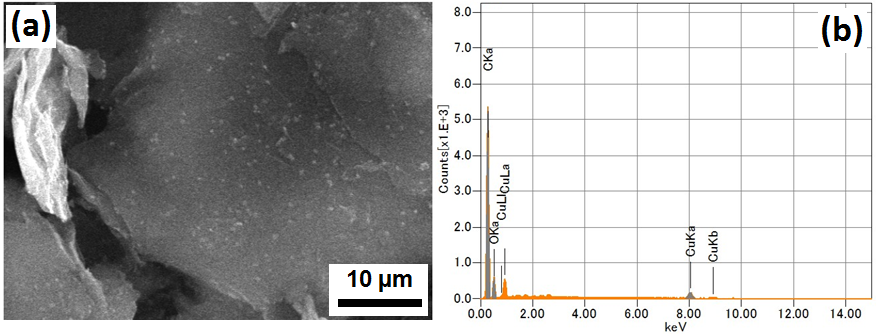


**Fig. S5**. (a) SEM image and (b) EDS spectrum of Cu-oxide/GO-I.


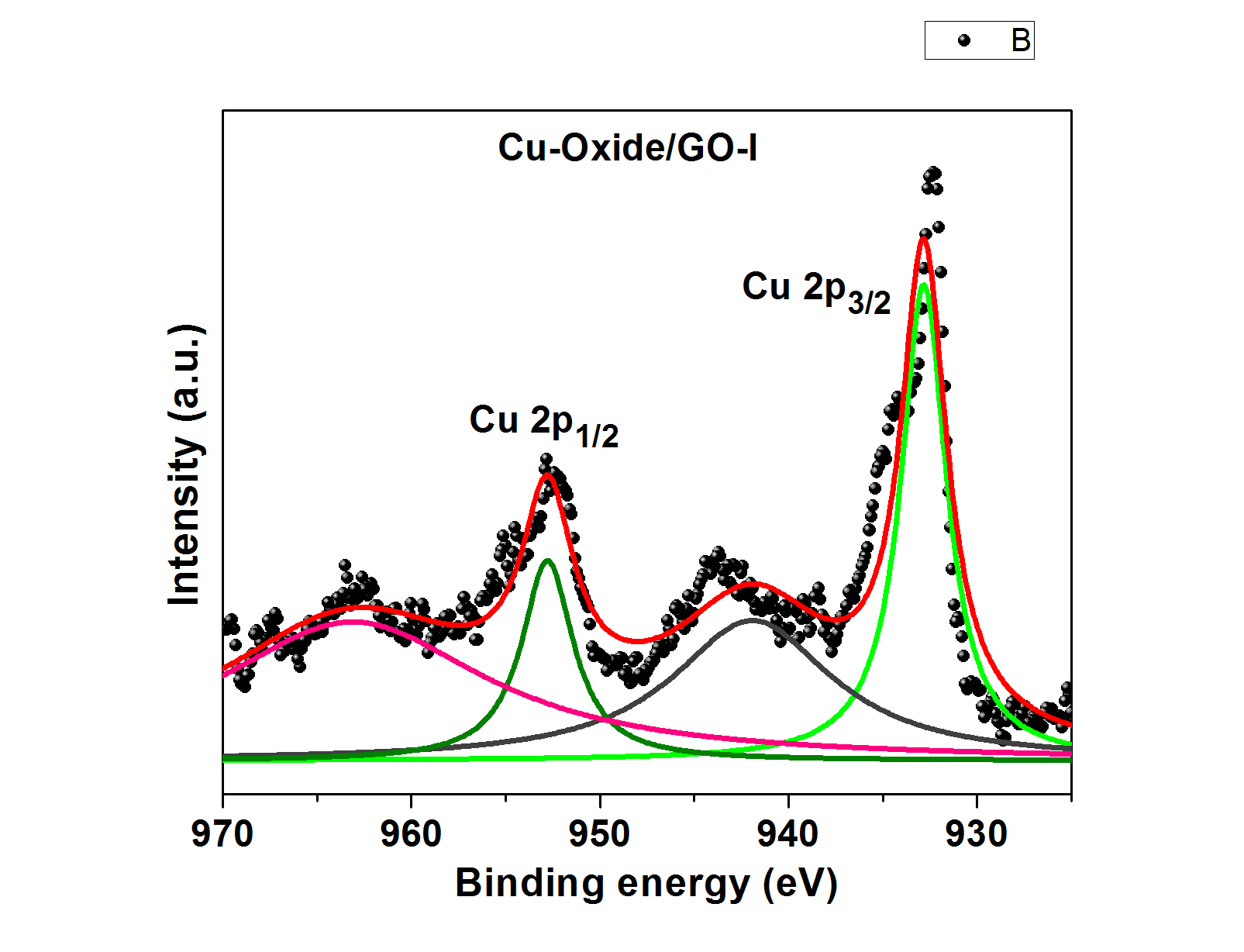


**Fig. S6**. Deconvoluted XPS Cu 2p spectrum of Cu-oxide/GO-I.


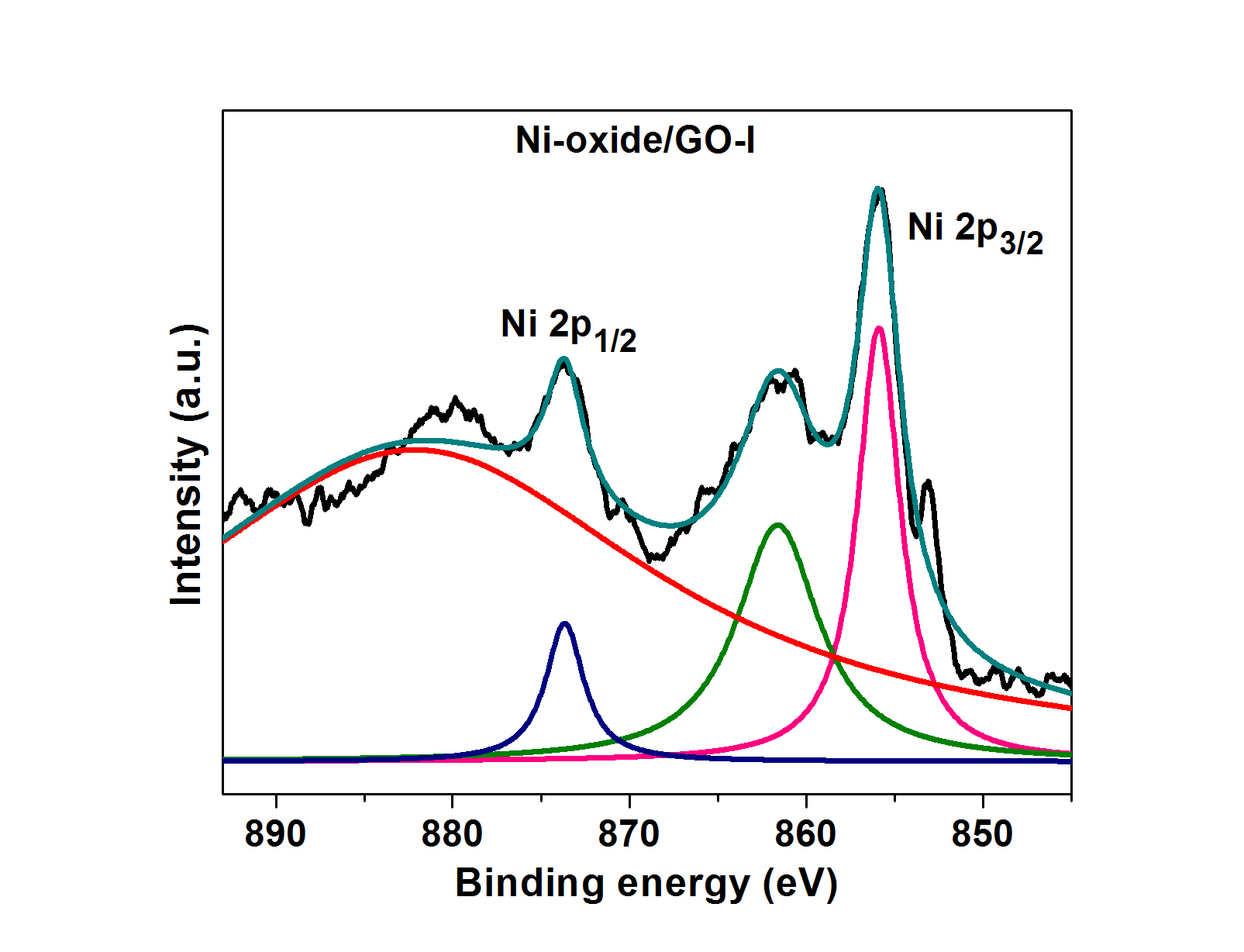


**Fig. S7**. Deconvoluted XPS Ni 2p spectrum of Ni-oxide/GO-I.

**NMR Data of the catalytic products**

**Table 2, entries 1 and 10 (1,4-dicyanobenzene) – Fig. S8**

^1^H NMR (CDCl_3_, 500 MHz): d 7.80 (s, 4H). ^13^C NMR (CDCl_3_, 125 MHz): d 116.7, 116.9, 132.7.

**Table 2, entries 2, 4 and 6 (benzonitrile) – Fig. S9**

^1^H NMR (CDCl_3_, 500 MHz): d 7.64-7.29 (m, 5H). ^13^C NMR (CDCl_3_, 125 MHz): d 132.8, 132.1, 129.1, 118.8, 1120.3.

**Table 2, entry 5 (4-pyridinecarbonitrile) - Fig. S10**

^1^H NMR (CDCl_3_, 500 MHz): d 7.80 (s, 4H). ^13^C NMR (CDCl_3_, 125 MHz): d 116.7, 116.9, 132.7.

**Table 2, entries 11 and 12 (4-chlorobenzonitrile) - Fig. S11**

^1^H NMR (CDCl_3_, 500 MHz): d 7.60 (d, *J* = 8.0 Hz, 2H), 7.47 (d, *J* = 8.0 Hz, 2H). ^13^C NMR (CDCl_3_, 125 MHz): d 139.5, 133.3, 129.7, 117.9, 110.8.


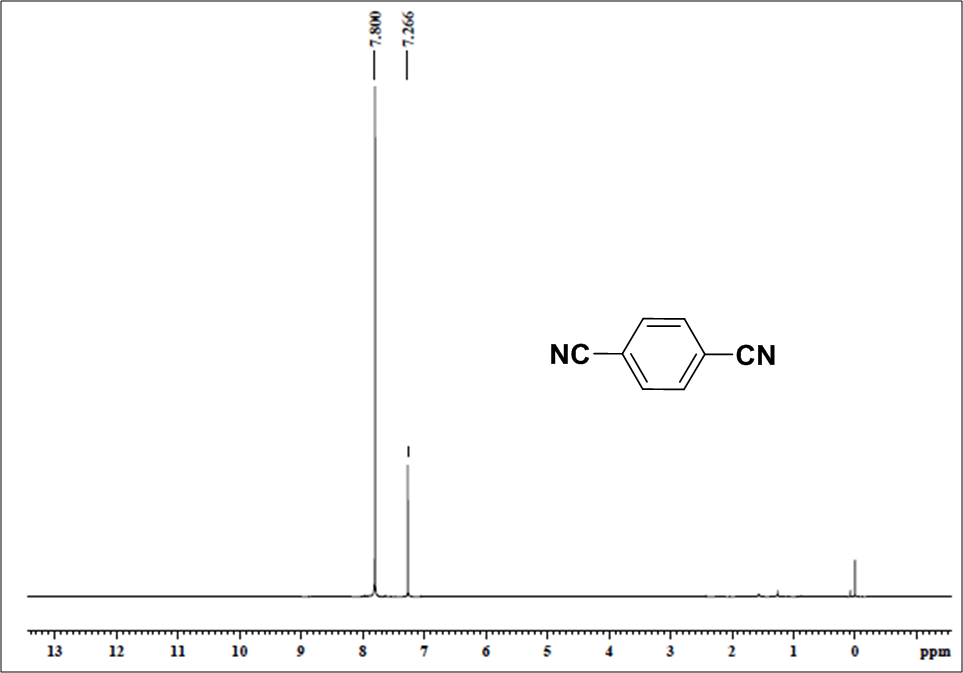


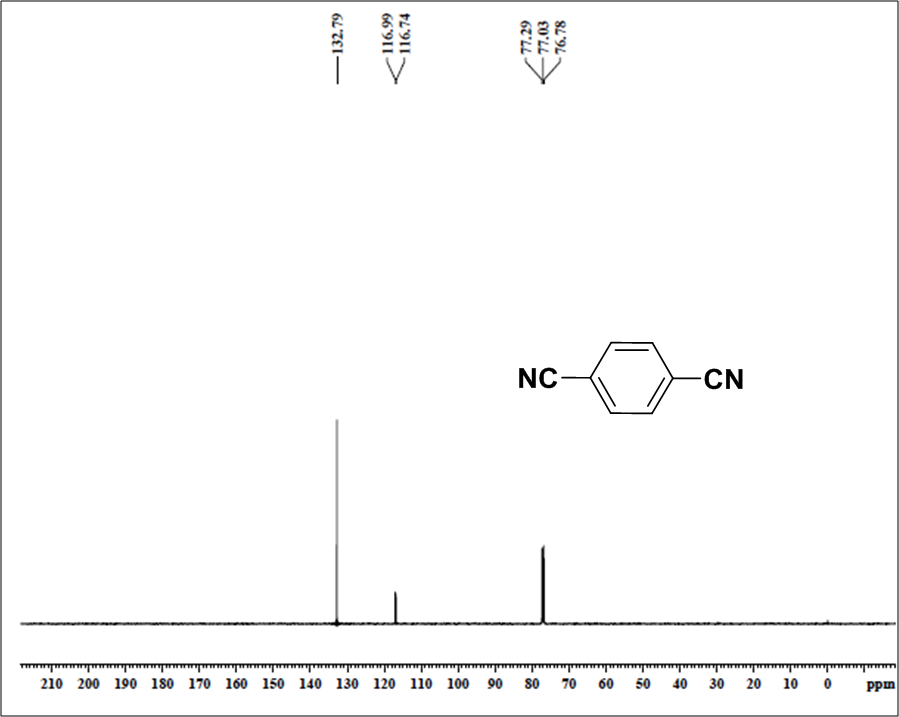


**Fig. S8.** ^1^H NMR and ^13^C NMR spectra of 1,4-dicyanobenzene (Table 2, entries 1 and 10).


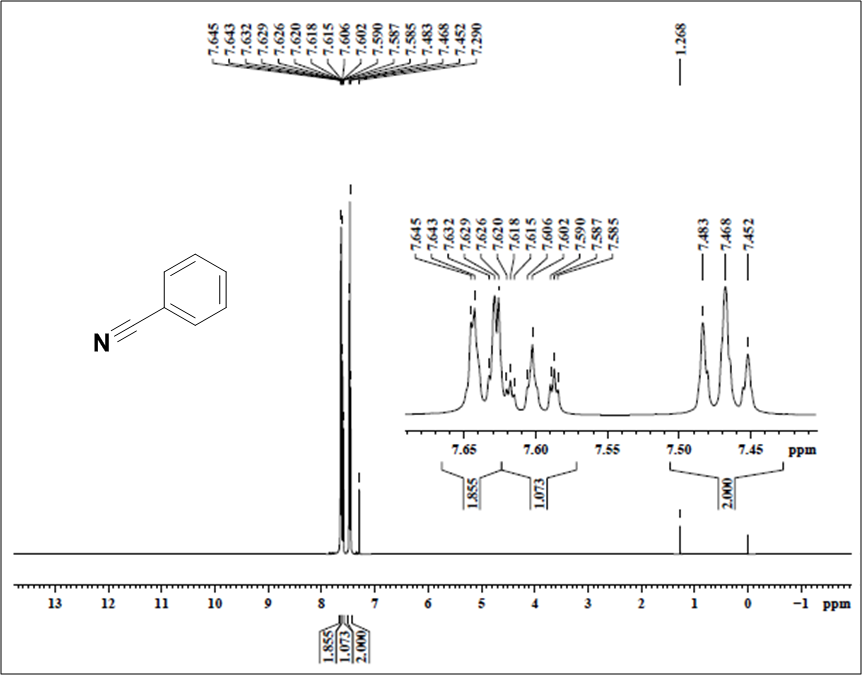


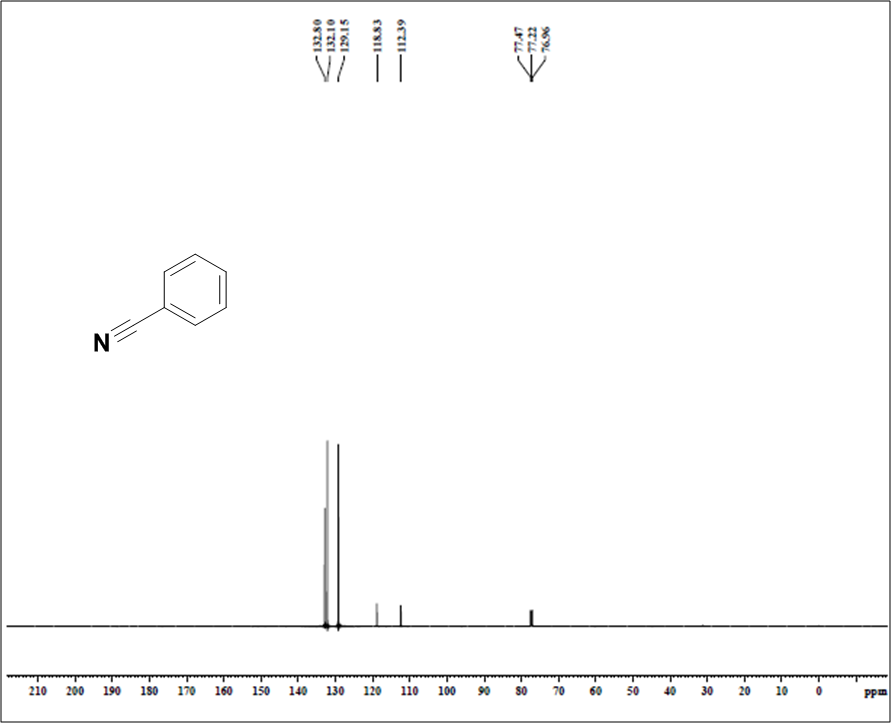


**Fig. S9**. ^1^H NMR and ^13^C NMR spectra of benzonitrile (Table 2, entries 2, 4 and 6).


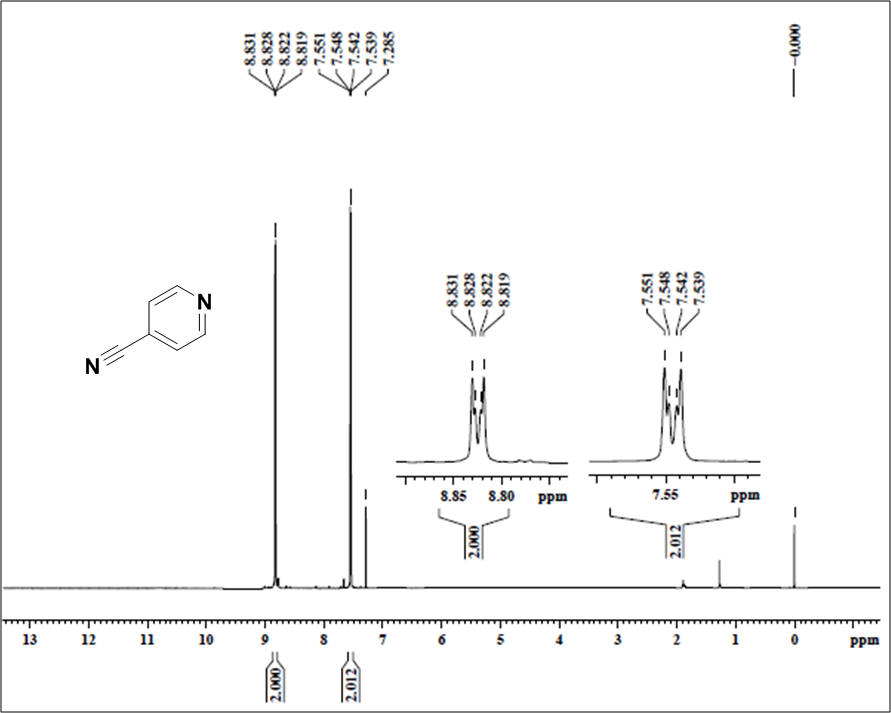


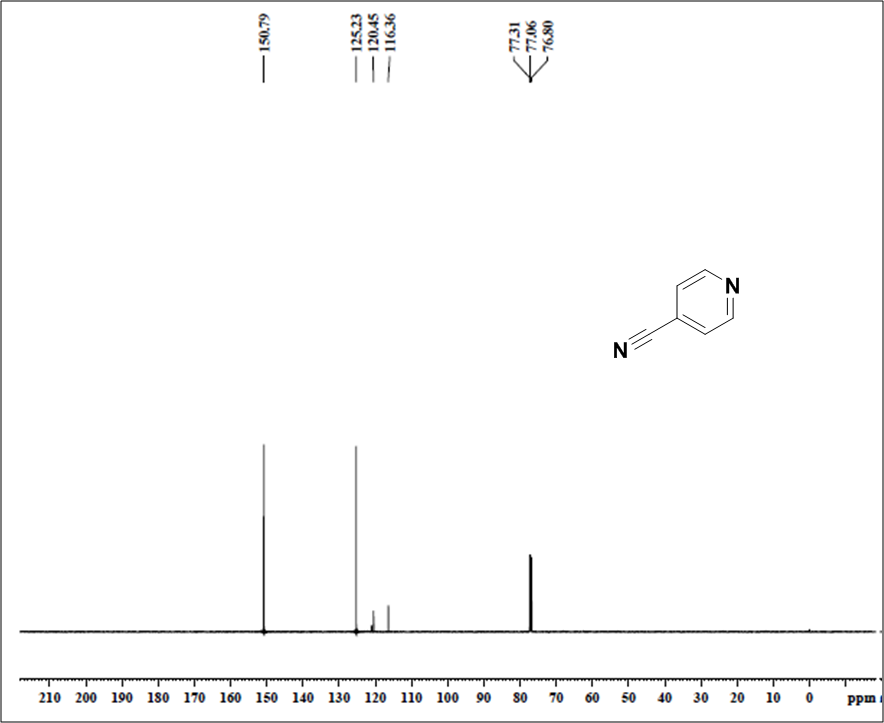


**Fig. S10.** ^1^H NMR and ^13^C NMR spectra of 4-pyridinecarbonitrile (Table 2, entry 5).


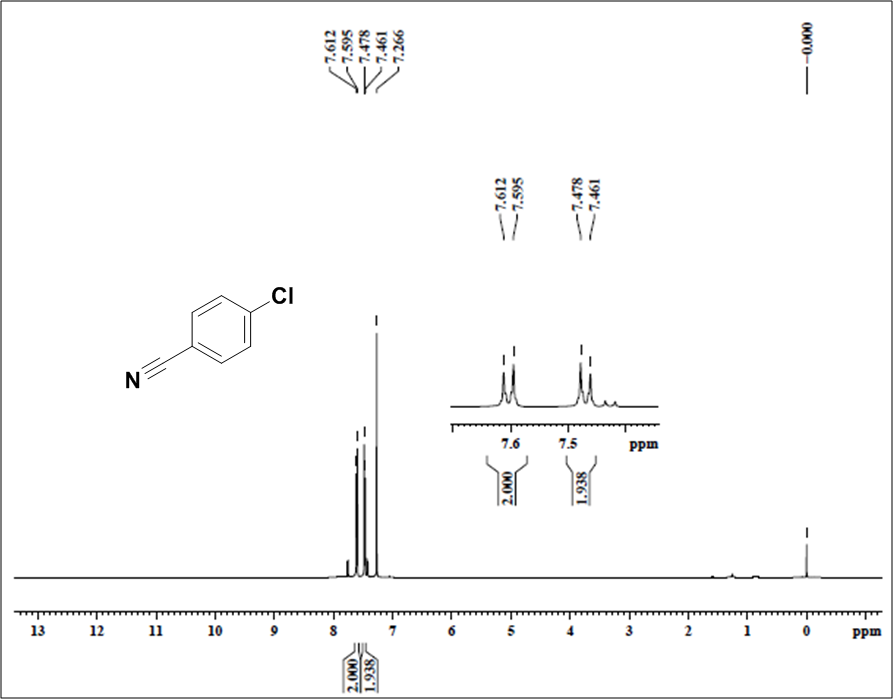


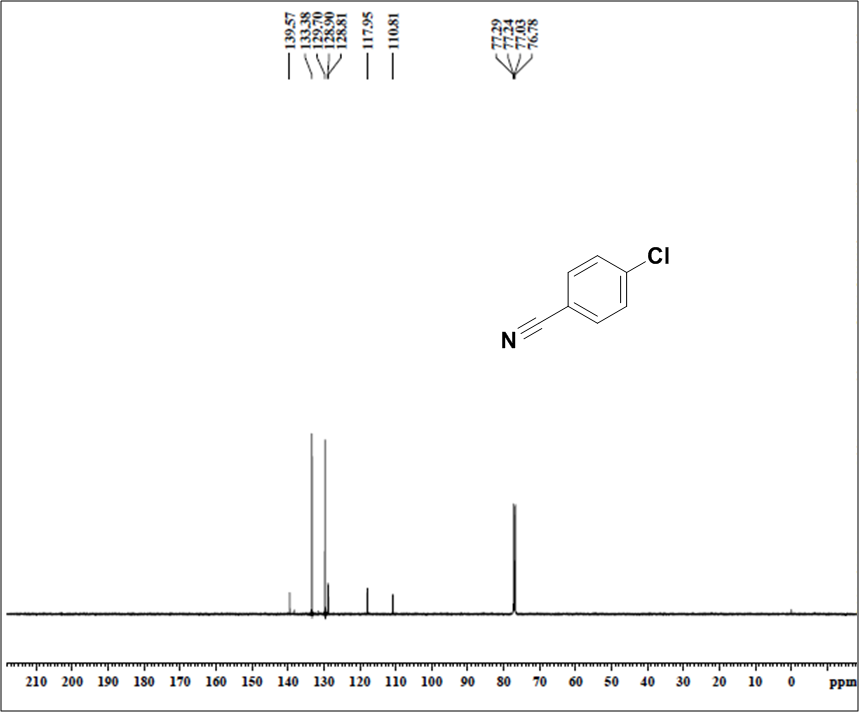


**Fig. S11**. ^1^H NMR and ^13^C NMR spectra of 4-chlorobenzonitrile (Table 2, entries 11 and 12).
